# The potential of artificial intelligence in enhancing adult weight loss: a scoping review

**DOI:** 10.1017/S1368980021000598

**Published:** 2021-06

**Authors:** Han Shi Jocelyn Chew, Wei How Darryl Ang, Ying Lau

**Affiliations:** Alice Lee Centre for Nursing Studies, Yong Loo Lin School of Medicine, National University of Singapore, 10 Medical Dr, Singapore 117597, Singapore

**Keywords:** Artificial intelligence, Obesity, Weight, Behaviour change, Self-regulation, Self-control, Diet

## Abstract

**Objective:**

To present an overview of how artificial intelligence (AI) could be used to regulate eating and dietary behaviours, exercise behaviours and weight loss.

**Design:**

A scoping review of global literature published from inception to 15 December 2020 was conducted according to Arksey and O’Malley’s five-step framework. Eight databases (CINAHL, Cochrane–Central, Embase, IEEE Xplore, PsycINFO, PubMed, Scopus and Web of Science) were searched. Included studies were independently screened for eligibility by two reviewers with good interrater reliability (*k* = 0·96).

**Results:**

Sixty-six out of 5573 potential studies were included, representing more than 2031 participants. Three tenets of self-regulation were identified – *self-monitoring* (*n* 66, 100 %), *optimisation of goal setting* (*n* 10, 15·2 %) and *self-control* (*n* 10, 15·2 %). Articles were also categorised into three AI applications, namely *machine perception* (*n* 50), *predictive analytics only* (*n* 6) and *real-time analytics with personalised micro-interventions* (*n* 10). Machine perception focused on recognising food items, eating behaviours, physical activities and estimating energy balance. Predictive analytics focused on predicting weight loss, intervention adherence, dietary lapses and emotional eating. Studies on the last theme focused on evaluating AI-assisted weight management interventions that instantaneously collected behavioural data, optimised prediction models for behavioural lapse events and enhance behavioural self-control through adaptive and personalised nudges/prompts. Only six studies reported average weight losses (2·4–4·7 %) of which two were statistically significant.

**Conclusion:**

The use of AI for weight loss is still undeveloped. Based on the current study findings, we proposed a framework on the applicability of AI for weight loss but cautioned its contingency upon engagement and contextualisation.

In 2016, the WHO estimated that 39 % of the global adult population were overweight and predicted an increase to 50 % by 2030^(1,2)^. Excessive fat accumulation is a major public health concern that increases one’s risk of cardiometabolic multi-morbidity and mortality by up to two and twenty-three times, respectively^(3–5)^. Concurrently, the yearly cost of treating obesity and its consequential diseases was estimated to reach US$1·2 trillion by 2025^(6)^. While pharmacotherapy (e.g., orlistat) and surgical interventions (e.g., bariatric surgery) have been effective and prompt in inducing weight loss, individuals often experience subsequent weight regain due to poor lifestyle habits^(7)^. Therefore, cheaper and safer diet and exercise programmes remain the preferred method for weight loss where up to 55 % of weight loss programme participants could lose ≥5 % of their initial body weight within a year^(8)^. However, studies have shown that weight loss often culminates after 6 months and individuals often regain up to 100 % of their initial weight within 5 years^(9,10)^. Failure to sustain weight loss has been attributed to the poor adherence to behaviour change plans^(9)^, lack of motivation^(11)^, knowledge^(12)^, coping skills and self-efficacy^(13)^, and central to weight loss failure is the lack of self-regulation^(14,15)^.

Self-regulation refers to the self-monitoring and self-control of automatic thoughts, emotions and behaviours to achieve a long-term goal (e.g., weight loss)^(16)^. Common self-regulation strategies for behaviour change include identifying discrepancies between current behaviours and future goals^(17)^, self-monitoring of behaviour and behavioural outcomes^(18)^, action planning^(19)^, goal setting^(20)^, habit change^(21)^ and behavioural substitution^(22)^. However, as compared to old habits which are largely automatic and effortless, such strategies are intentional, effortful and cognitively demanding^(23)^. This often leads to the temporal erosion of behaviour change adherence, causing a well-known yo-yo weight effect (weight increases back to baseline)^(24)^. Therefore, individuals trying to lose weight often attempt to either increase self-regulation capacity through sheer willpower^(25)^ or reduce the self-regulation effort needed through weight-loss mobile apps^(26)^, clinical weight management programmes^(27)^ and commercial weight-loss programmes^(28)^. However, such methods are often expensive, resource-intensive and unsustainable^(29)^. An emerging strategy to tackle this problem of poor self-regulation is to apply artificial intelligence (AI)^(30)^.

AI refers to the mimicry of human intelligence through machine learning to attain and apply knowledge and skills for processes such as pattern recognition and decision-making. The popularity of AI stems from its potential to solve real-world problems with rationality, efficiency, cost-effectiveness and accuracy. In obesity research, AI has been used to examine aetiologies^(31)^, perform risk profiling^(32)^, standardise diagnosis (decision support system)^(33)^, personalise weight management programmes^(34)^, perform remote monitoring^(32)^ and predict prognoses^(35)^. However, to the authors’ best knowledge, there are limited academic publications that explored the use of AI to improve behaviour change self-regulation for weight loss^(36)^.

Therefore, we conducted a scoping review to present an overview of the possible applications of AI to regulate eating and dietary behaviours, exercise behaviours and weight loss. Unlike a systematic review that aims to answer a specific research question, a scoping review aims to map out the ‘breath, nature and extent of research’ done on a topic without dwelling into the literature or assessing its methodological quality^(37)^. This aims to provide a comprehensive collection of articles on a specific topic, elucidate research gaps in their underexplored aspects and inform the worth of conducting a systematic review. In 2017–2018, approximately 45 % of middle-aged adults (40–59 years old), 43 % of older adults and 40 % of younger adults were obese^(38)^. This indicates that weight management should begin at a younger age before the onset of obesity and chronic diseases, which commonly occurs during middle-age due to a slower metabolism, increased food consumption and an increasingly sedentary lifestyle^(39–41)^. Therefore, the literature search was narrowed down to adults from 18–64 years old to enhance the focus and clarity of this inquiry.

## Methods

This scoping review was structured according to the five-step framework by Arksey and O’Malley, and results were presented according to the Preferred Reporting Items for Systematic Reviews and Meta-Analyses extension for scoping reviews (PRISMA-ScR) guidelines (online supplementary material, Supplemental Table S1)^(42,43)^.

### Step 1: Identifying the research question

We used the Population, Intervention, Comparison and Outcomes (PICO) acronym to develop our research question, ‘what is known about the potential of AI for weight loss and weight-related behaviour change’.

### Step 2: Identifying relevant studies

Studies were first searched across eight electronic databases (CINAHL, Cochrane–Central, Embase, IEEE Xplore, PsycINFO, PubMed, Scopus and Web of Science) for papers published from inception till 22 July 2020. Initial search terms such as ‘artificial intelligence’ and ‘weight loss’ were iteratively derived from the PICO framework and medical subject heading through multiple rounds of database searching by the HSJC in consultation with LY. The final search terms used were ‘artificial intelligence’; ‘machine learning’; ‘computational intelligence’; ‘computer heuristics’; ‘expert system’; ‘fuzzy logic’; ‘knowledge bases’; ‘natural language processing’; ‘neural networks’; ‘weight loss’; ‘weight management’ and ‘weight control’ (see online supplementary material, Supplemental Table S2 for search terms used in different databases). Upon mapping the existing studies into three broad categories, we found that weight-related changes were centralised around diet and exercise. Therefore, we conducted another search for literature published up till 15 December 2020 using additional keywords such as ‘diet’, ‘eating’, ‘physical activity’, ‘sedentary’ and ‘exercise’.

### Step 3: Study selection

**Fig. 1:**
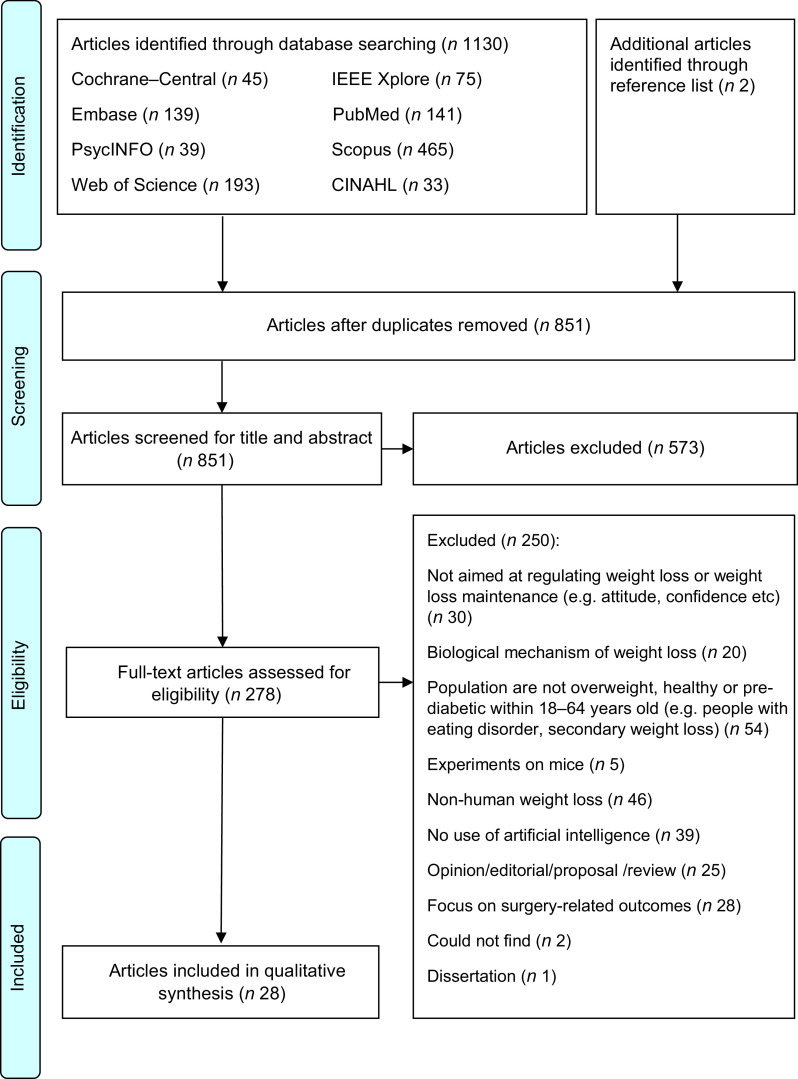
PRISMA 2009 flow diagram for first search

**Fig. 2:**
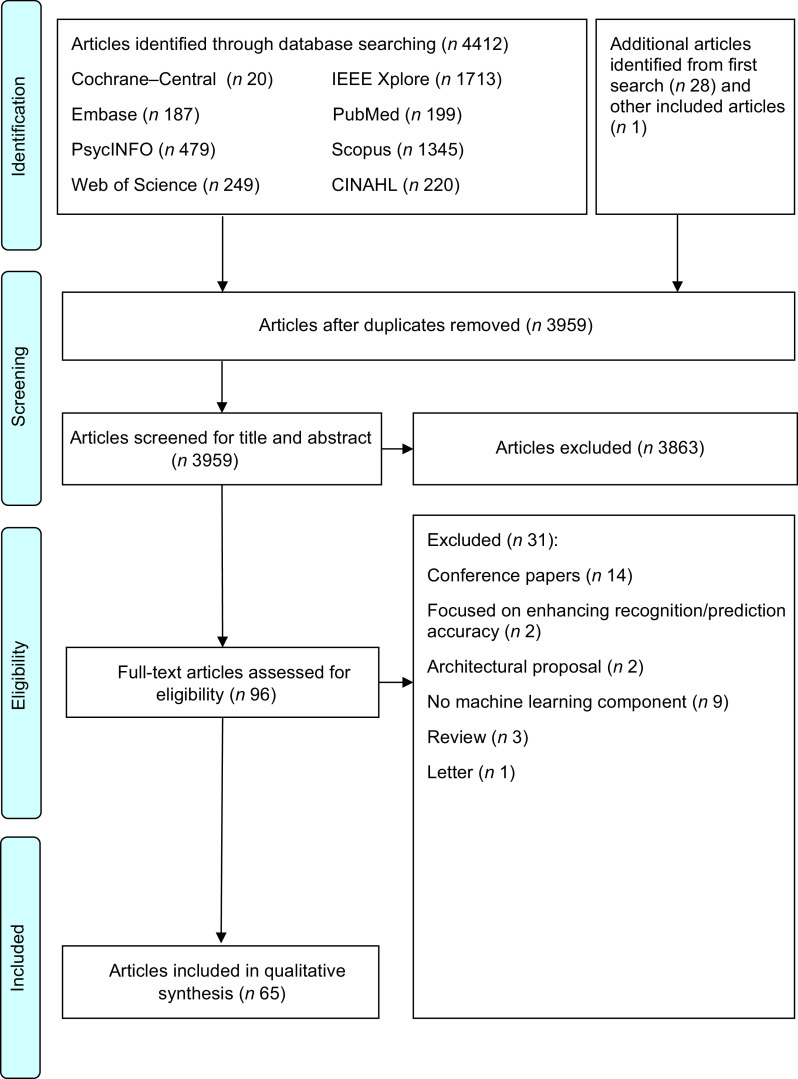
PRISMA 2009 flow diagram for second search

After the database searching, duplicate articles were removed and the remaining titles and abstracts were screened for eligibility. Full texts of the articles were independently screened for eligibility by HSJC and WHDA where discrepancies were resolved through discussions. Studies were included if they described the use of AI for weight loss or weight loss-related behaviour change in adults aged 18–64 years. Studies were excluded if they: (1) did not describe the use of AI (e.g., purely data scraping); (2) were grey literature including conference, opinion, protocol or technical/theoretical papers; (3) were on people undergoing surgery (e.g., bariatric surgery) or with underlying diseases (excluding pre-diabetes) that affect weight status; (4) were unrelated to self-regulation and (5) were not written in the English language. Additional studies were then identified using forward and backward reference searching of the included articles. The search process and results are shown in Figs 1 and 2.

### Step 4: Charting the data

**Fig. 3:**
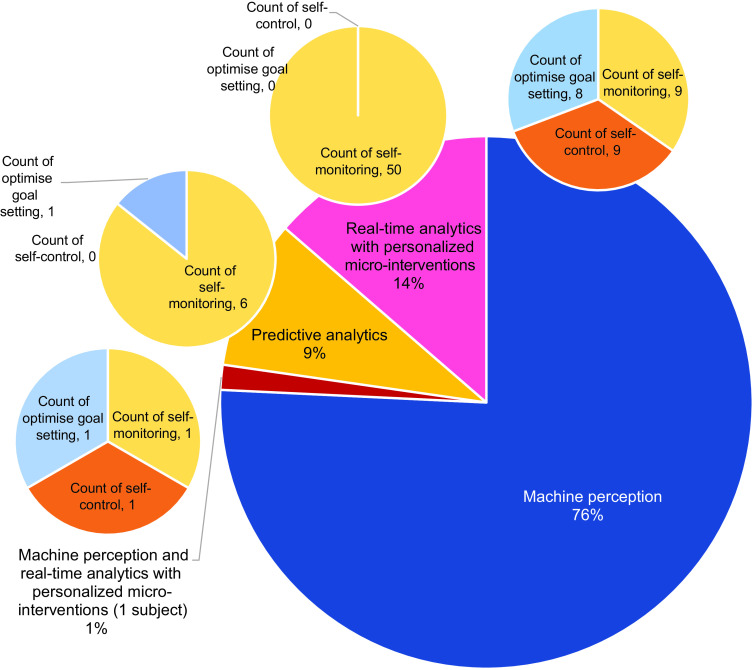
Data mapping of AI features used for different self-regulation components (*n* 66)

Data extraction was performed according to a form developed by HSJC, which was pilot tested on five articles and refined accordingly before use. Information extracted was categorised under the headers – author, year, country, type of publication, study design, aim, population, sample size, age, sex, BMI, self-regulation tenets (e.g., self-monitoring), AI functions (e.g., recognise eating behaviours), AI features (e.g., gesture recognition and predictive analytics), weight loss-related behaviours (e.g., dieting), machine learning techniques, data collection methods and important results. The resultant information was then charted as shown in Fig. 3.

## Step 5: Collating, summarising and reporting the results

### Study characteristics

As shown in Fig. 1, 1132 potential articles were retrieved from the first database search, 851 titles and abstracts were screened, 278 full-text articles were assessed and twenty-eight articles were included. As shown in Fig. 2, 4441 articles were retrieved from the second database search, 3959 titles and abstracts were screened, ninety-six full-text articles were assessed and sixty-five articles were included. The kappa statistic (*k*) indicated good interrater reliability (*k* = 0·96) where discrepancies were resolved upon discussion. During the screening of full-text articles, two articles were unable to be retrieved even after seeking help from the university librarian and hence were excluded^(44,45)^. Two separate journal articles included in this review were published from the same dissertation^(46–48)^. Among the sixty-five included articles, one reported two studies and hence a total of sixty-six studies were presented in this scoping review. Representing more than 2031 participants, 56·1 % of the studies were from the USA, 87·9 % were experimental studies, 81·8 % had a sample size of < 100 participants, 89·4 % included participants from both sexes and 56·1 % reported the baseline BMI of the participants (Table 1). Study characteristics are detailed in online supplementary material, Supplemental Table S3.

**Table 1:** Study characteristics (*n* 66)*

Study characteristics	*n*	%
Country of study		
USA	37	56·1
Canada	3	4·5
Finland	2	3·0
France	1	1·5
Germany	1	1·5
Greece	1	1·5
Switzerland	2	3·0
The Netherlands	1	1·5
UK	5	7·6
China (including one study from Hong Kong)	6	9·1
South Korea	2	3·0
Taiwan	2	3·0
India	1	1·5
Saudi Arabia	1	1·5
Australia	1	1·5
Study design		
RCT	8	12·1
Experimental (lab-based, quasi-experimental, pretest-posttest)	50	75·8
Observational	6	9·1
Secondary data analysis	2	3·0
Sample size		
< 100	54	81·8
100–300	5	7·6
Not reported	8	12·1
Sex		
All females	1	1·5
All males	0	0
Both sexes	59	89·4
Not reported	7	24
Mean age (years old):		
20–30	19	28·8
31–40	7	10·6
41–50	3	4·5
50–60	5	7·6
Not reported†	28	42·4
Baseline BMI (kg/m^2^)		
Reported	37	56·1
Not reported	30	45·5

* One included article consisted of two studies; hence, the total number of studies is 66.

† Includes ten studies that provided age ranges.

### Self-regulation of weight loss-related behaviours

Three tenets of self-regulation were identified, namely *self-monitoring* (*n* 66, 100 %), *optimisation of goal setting* (*n* 10, 15·2 %)^(26,49–55)^ and *self-control* (*n* 10, 15·2 %)^(26,49–51)^. Details on the use of AI for the self-regulation of weight loss-related behaviours are shown in Table 2. Of the studies on enhancing *self-monitoring*, twenty-nine (43·9 %) were on eating behaviours^(58–76)^, seven (10·6 %) were on energy intake^(34, 77–82)^, thirty-three (50 %) were on physical activity^(26,51–55,60,74,81–105)^ and nine (13·6 %) were on energy expenditure^(83,85,92,94–97,100,101)^. Of the studies on *optimising goal setting*, five were on optimising eating behaviour goals (e.g., eating at a certain time of the day and energy intake)^(48,49,53)^ and six were on optimising physical activity goals (e.g., type of physical activity and energy expenditure)^(26,51–55)^. Of the ten studies on *self-control*, five were on controlling eating behaviours^(48,49,107)^, three were on controlling physical activity performance^(26,52,54)^ and two were on both^(51,53)^. Only six of these studies reported weight loss of which two were significant^(26,107)^. With only 15·2 % of the included studies examining strategies to exert self-control over weight-related behaviours, more research is needed to explore the potential of AI on improving weight-related behavioural changes for weight loss.

**Table 2:** Functions of AI in self-regulation of weight management in healthy and overweight populations (*n* 66)

Author, year	Self-regulation use case	AI features	AI functions	Data collection instrument	Data type	Important results
Alshurafa *et al.*, 2015	Self-monitoring (eating behaviour)	Gesture recognition	To distinguish between food types	Necklace piezoelectric sensor (vibration sensor)	Skin motion when swallowing	Successfully distinguished between liquids and solids (*F*-measure > 90 %), hot and cold drinks (*F*-measure > 90 %) and between solid food types (*F*-measure ∼80 %)
Amft *et al.*, 2008	Self-monitoring (eating behaviour)	Gesture and sound recognition	To recognise dietary activities using on-body sensors	On-body sensors: (1) to detect arm movements: inertial motion sensors (three-dimensional acceleration, gyroscope and magnetometers) at the wrist and upper back (integrated into a jacket); (2) to detect chewing: ear microphone located inside the ear canal to detect bone-conducted food breakdown sounds and (3) to detect swallowing: collar sensor containing surface electromyography (EMG) electrodes and a stethoscope microphone	Arm movements, chewing cycle sounds and swallowing	Four intake gestures from arm movements and two food groups from chewing cycle sounds were detected and identified with a recall of 80–90 % and a precision of 50–64 %. The detection of individual swallows resulted in 68 % recall and 20 % precision. Sample-accurate recognition rates were 79 % for movements, 86 % for chewing and 70 % for swallowing
Amft *et al.*, 2009	Self-monitoring (eating behaviour)	Sound recognition	To evaluate the prediction of food weight in individual bites using an ear-pad chewing sound sensor	Ear-pad chewing sound sensor	Chewing cycles and food type	Sound-based chewing recognition achieved recalls of 80 % at 60–70 % precision. Food classification of chewing sequences had an average accuracy of 94 %. Mean weight prediction error was lowest for apples (19·4 %) and the largest for lettuce (31 %)
Arif *et al.*, 2017	Self-monitoring (physical activity)	Gesture recognition	Recognise physical activity type	Rotation forest classifier	Inertial measurement units (IMUs) placed at wrist, chest and ankles	Accurately (98 %) identified seventeen physical activities ranging from ambulation to activities of daily living
Aswani *et al.*, 2019	Self-monitoring (eating behaviour; physical activity), optimise goal setting	Predictive analytics	To predict weight loss based on subject characteristics, step count, energy intake and counselling sessions	Bayesian classification	Secondary data	Predictive modelling framework was competitive in terms of prediction accuracy with linear SVM, logistic regression and decision tree models, which further justified the use of the utility-maximising framework and its ability to capture ‘irrational’ discounting in the decision-making of individuals participating in the intervention
Aziz *et al.*, 2020	Self-monitoring (physical activity and energy expenditure)	Gesture recognition	To estimate energy expenditure during sitting, standing and treadmill walking using a smartwatch	LG Urbane Android smartwatch: tri-axial accelerometer, gyroscope and magnetometer	Physical movement	The activity-based models provided 7 % better energy expenditure estimation than the traditional acceleration-based models
Bastian *et al.*, 2015	Self-monitoring (physical activity)	Gesture recognition	To discriminate between eight activity classes (lying, slouching, sitting, standing, walking, running and cycling) in a laboratory condition and walking the streets, running, cycling and taking the bus in free-living conditions	Hip-worn triaxial accelerometer	Physical movement	The performances of the laboratory-calibrated algorithm decreased for several activities when applied to free-living data. Recalibrating the algorithm with data closer to real-life conditions improved the detection of overall sitting (sensitivity: laboratory model 24·9 %; recalibrated model 95·7 %).
Bi *et al.*, 2016	Self-monitoring (eating behaviour)	Sound recognition	To monitor and recognise food intakes in daily life	High-fidelity microphone is worn on the subject’s neck near the jaw	Acoustic signals	The accuracy of food-type recognition by AutoDietary is 84·9 %, and those to classify liquid and solid food intakes are up to 97·6 and 99·7 %, respectively.
Bouarfa *et al.*, 2014	Self-monitoring (physical activity, energy expenditure)	Gesture recognition	Energy expenditure estimation under free-living conditions	A single ear-worn activity recognition (eAR) sensor (built-in triaxial accelerometer)	Physical movement	In free-living settings, ten different types of physical activities (i.e., lying down, standing, computer work, vacuuming, going up and downstairs, slow walking, brisk walking, slow running, fast running and cycling) were predicted
Chung *et al.*, 2018	Self-monitoring (eating behaviour and physical activity)	Gesture recognition	To detect the patterns of temporalis muscle activities during food intake and other physical activities	A glasses-type device with an in-built EMG or piezoelectric strain sensor and attaching them directly onto human skin	Skin movement	The average F1 score of the classification among the featured activities was 91·4 %.
Dijkhuis *et al.*, 2018	Self-monitoring (physical activity), optimise goal setting	Predictive analytics	To predict the likelihood of a user to achieve a daily personal step goal	Wrist-worn activity tracker, the Fitbit Flex	Physical activity	In 80 % of the individual cases, the random forest algorithm was the best performing algorithm
Dobbins *et al.*, 2017	Self-monitoring (physical activity)	Gesture recognition	To distinguishing physical activity	Tri-axial accelerometers and a heart-rate monitor	Physical movement	The results showed an improvement in recognition accuracy as compared with existing studies, with accuracies of up to 99 % and sensitivities of 100 %
Dong *et al.*, 2014	Self-monitoring (eating behaviour)	Gesture recognition	To automatically detect periods of eating (free-living condition)	iPhone 4 (accelerometer and gyroscope) placed inside a pouch, wrapped snugly around the forearm	Wrist motion (linear and rotational)	Results show an accuracy of 81 % for detecting eating at 1 s resolution
Ermes *et al.*, 2008	Self-monitoring (physical activity)	Gesture recognition	To recognise sports performed by the subjects	3-D accelerometers on hip and wrist and GPS information	Physical movement	The total accuracy of the activity recognition using both supervised and unsupervised data was 89 % that was only 1 % unit lower than the accuracy of activity recognition using only supervised data. The accuracy decreased by 17 % unit when only supervised data were used for training and only unsupervised data for validation.
Everett *et al.*, 2018	Self-monitoring (physical activity), self-control (physical activity), optimise goal setting	Real-time analytics with personalised micro-interventions	Automatically translate various raw mobile phone data into insights about user’s life habits. Provide personalised, contextual, just-in-time, just-in place recommendations (tailored messages)	Phone accelerometer	Physical movement	Physical activity increased by 2·8 metabolic equivalents of task (MET) – hours per week, weight reduced by 1·6 kg (*P* < 0·001) (˜2 %). BMI declined by 0·6 kg/m^2^ (*P* < 0·001), and waist circumference was reduced by 1·4 cm (*P* < 0·01)
Fontana *et al.*, 2014	Self-monitoring (eating behaviour)	Gesture recognition	To objectively monitor ingestive behaviour in free-living	A jaw motion sensor, a hand gesture sensor and an accelerometer (integrated into a device and wirelessly interface to a smartphone)	Jaw and hand motion	The system was able to detect food intake with an average accuracy of 89·8 %
Forman *et al.*, 2018	Self-monitoring (eating behaviour), self-control (eating behaviour), optimise goal setting	Real-time analytics with personalised micro-interventions	Predict dietary lapses and deliver a targeted intervention designed to prevent the lapse from occurring	Ensemble methods (combining weighted vote of predictions from random forest, Logit. Boost, Bagging, Random Subspace, Bayes Net)	Ecological momentary assessment (EMA) 6 times a day + ad hoc entry of lapse event	Of the twenty-one possible triggers, 29 % of intervention triggers were based on time of day, 16·7 % based on low motivation and 10 % based on fatigue. The remaining eighteen triggers were identified as risk factors < 10 % of the time. There was a reduction in unplanned lapses. Participants averaged a 3·13 % weight loss
Forman *et al.*, 2019a	Self-monitoring (eating behaviour), self-control (eating behaviour), optimise goal setting	Real-time analytics with personalised micro-interventions	Predict dietary lapses and deliver a targeted intervention designed to prevent the lapse from occurring	Ensemble methods (e.g., combining weighted vote of predictions from Random Forest, Logit. Boost, Bagging, Random Subspace, Bayes Net)	EMA 6 times a day + ad hoc entry of lapse event	Weight Watcher (WW) + OnTrack (OT) participants reported an average of 29·72 (sd = 29·11) lapses during the 10-week study period, and the frequency decreased through time. Weight losses were greater for WW + OT (M = 4·7 %, se = 0·55) than for WW (M = 2·6 %, se = 0·80)
Forman *et al.*, 2019b	Self-monitoring (eating behaviour), self-control (eating behaviour), optimise goal setting	Real-time analytics with personalised micro-interventions	Predict dietary lapses and deliver a targeted intervention designed to prevent the lapse from occurring	Reinforcement learning algorithm	Physical activity measured in minutes of moderate-to-vigorous physical activity (MVPA) using Fitbit Flex or a similar type of Fitbit wrist-worn activity tracker	Proposed system achieved weight losses equivalent to existing human coaching programmes (non-optimised (NO) = 4·42 %, individually optimised (IO) = 4·56 %, group-optimised (GO) = 4·39 %) at roughly one-third the cost (1·73 and 1·77 coaching hours/participant for IO and GO, *v*. 4·38 for NO)
Fullerton *et al.*, 2017	Self-monitoring (physical activity)	Gesture and image recognition	To recognise activity and sub-category activity types through the use of multiple body-worn accelerometers in a free-living environment	Nine body-worn accelerometers for a day of free living	Physical movement	The recognition accuracy of 97·6 %. Controlled and free-living testing provided highly accurate recognition for sub-category activities (> 95·0 %). Decision tree classifiers and maximum features demonstrated to have the lowest computing time
Goldstein *et al.*, 2018	Self-monitoring (eating behaviour)	Predictive analytics	Predict dietary lapses	Ensemble methods (combining weighted vote of predictions from random forest, Logit. Boost, Bagging, Random Subspace, Bayes Net)	EMA 6 times a day + ad hoc entry of lapse event	Participants responded to an average of 94·6 % of EMA prompts (range = 85·2–98·9 %) and compliance remained relatively stable throughout the study
Goldstein *et al.*, 2020	Self-monitoring (eating behaviour), self-control (eating behaviour) optimise goal setting	Real-time analytics with personalised micro-interventions	To measure dietary lapses and relevant lapse triggers and provide personalised intervention using machine learning	Decision tree	EMA 6 times a day + ad hoc entry of lapse event	Average of 4·36 lapses per week (sd = 1·46). Participants lost an average of 2·6 % of their starting weight at mid-treatment and 3·4 % at end-of-treatment
Hegde *et al.*, 2017	Self-monitoring (physical activity)	Gesture recognition	To propose an insole-based activity monitor – SmartStep, designed to be socially acceptable and comfortable	Insole-based sensor system: contains a 3D accelerometer, a gyroscope The wrist sensor was worn on the wrist of the dominant hand like a wristwatch The ActivPal (AP), a commercially available positional sensor module worn on the thigh as a criterion measure during the free-living study. It classifies individuals’ activities into periods spent sedentary, standing and stepping	Physical movement	The overall agreement with ActivPAL was 82·5 % (compared with 97 % for the laboratory study). The SmartStep scored the best on the perceived comfort reported at the end of the study
Hezarjaribi *et al.*, 2018	Self-monitoring (energy intake)	Speech recognition	To facilitate nutrition monitoring using speech recognition and text mining	Smartphone microphone	Speech	Speech2Health achieves an accuracy of 92·2 % in computing energy intake
Hossain *et al.*, 2020	Self-monitoring (eating behaviour)	Image recognition	To detect and count bites and chews automatically from meal videos	Video recorder	Video images	Mean accuracy of 85·4 % ± 6·3 % concerning manual annotation was obtained for the number of bites and 88·9 % (± 7·4 %) for the number of chews
Hua *et al.*, 2020	Self-monitoring (physical activity)	Gesture recognition	To classify nine different upper extremity exercises	Triaxial IMU	Physical movement (kinematics)	Random forest models with flattened kinematic data as a feature had the greatest accuracy (98·6 %). Using the triaxial joint range of motion as the feature set resulted in decreased accuracy (91·9 %) with faster speeds
Huang *et al.*, 2017	Self-monitoring (eating behaviour)	Gesture recognition	Recognise eating behaviour and food type	On-board real-time decision algorithm; chewing detection algorithm; decision trees	Electromyography embedded in wearable glasses connected to the smartphone	96 % accuracy in detecting chewing and classifying five types of food
Jain *et al.*, 2018	Self-monitoring (physical activity)	Gesture recognition	To classify activities using built-in sensors of smartphones	The phone kept in the front pocket of the subject’s trousers, built-in accelerometer and gyroscope sensor	Physical movement	Average activity classification accuracy achieved using the proposed method was 97·12 %
Jiang *et al.*, 2020	Self-monitoring (energy intake)	Image recognition	To develop a deep model-based food recognition and dietary assessment system to study and analyse food items from daily meal images (e.g., captured by smartphone)	Existing datasets	Images	The system was able to recognise food items accurately with top-1 accuracy of 71·7 % and top-5 accuracy of 93·1 %
Juarascio *et al.*, 2020	Self-monitoring (eating behaviour)	Predictive analytics	To detect changes in HRV to in turn detect the risk of experiencing an emotional eating episode in an ecologically valid setting	Empatica E4 wrist-sensor (photoplethysmography: non-invasive the optical measurement that can derive cardiovascular features from light absorption of the skin) and EMA six prompts per day (participants were also instructed to self-report immediately following an emotional eating episode and answer the same questions)	Heart rate variability (HRV)	Support vector machine (SVM) models using frequency-domain features achieved the highest classification accuracy (77·99 %), sensitivity (78·75 %) and specificity (75·00 %), though were less accurate at classifying episodes (accuracy 63·48 %, sensitivity 62·68 % and specificity 70·00 %) and did not meet acceptable classification accuracy
Kang *et al.*, 2019	Self-monitoring (physical activity, energy expenditure)	Gesture recognition	To predict the energy expenditure of physical activities	AirBeat system: built-in patch-type sensor module for wireless monitoring of heart rate, exercise index, ECG and a three-axial acceleration motion detector	Physical movement, HR, exercise index, ECG, humidity, and temperature	RMSE of 0·1893 and *R* ^2^ of 0·91 for the energy expenditures of aerobic and anaerobic exercises
Kim *et al.*, 2015	Self-monitoring (physical activity)	Gesture and image recognition	To recognise sedentary behaviour	Two accelerometers (waist over the right hip and right thigh) and a wearable camera (around the neck using a lanyard)	Physical movement	ActivPAL showed the most accurate estimate of total sedentary time with MAPE of 4·11 % and percentage of bias of –3·52 %
Korpusik *et al.*, 2017	Self-monitoring (energy intake)	Speech recognition	To automatically extract food concepts (nutrients and energetic intake) from a user’s spoken meal description	Amazon Mechanical Turk (AMT) where Turkers were to record ten meal descriptions	Speech	83 % semantic tagging accuracy
Kyritsis *et al.*, 2019	Self-monitoring (eating behaviour)	Gesture recognition	To automatically detect in-meal food intake cycles using the inertial signals (acceleration and orientation velocity) from an off-the-shelf smartwatch	Off-the-shelf smartwatch (acceleration and orientation velocity)	In-meal bite detection	Achieved the highest F1 detection score (0·913 in the leave-one subject-out experiment) as compared with existing algorithms
Lin *et al.*, 2012	Self-monitoring (physical activity, energy expenditure)	Gesture recognition	To recognise physical activities and their corresponding energy expenditure	Motion sensors and an ECG sensor	Physical movement and ECG	Recognition accuracies using decision trees in the cross-validations ranged from 95·52 to 97·70 %
Lin *et al.*, 2019	Self-monitoring (physical activity, energy expenditure)	Image recognition	To estimate energy expenditure of physical activity in gyms	Kinect for XBOX 360 sensors (depth and motion sensing)	Physical movement (Kinect skeletal data)	The measured and predicted metabolic equivalents of task exhibited a strong positive correlation
Liu *et al.*, 2012	Self-monitoring (physical activity, energy expenditure)	Gesture recognition	To recognise the physical activity	Two triaxial accelerometers (at hip and wrist), and one ventilation sensor secured to the abdomen (AB) at the level of the umbilicus	Ventilation (abdomen), motion (hip), motion (wrist)	Correctly recognised the thirteen activity types 88·1 % of the time, which is 12·3 % higher than using a hip accelerometer alone. Also, the method predicted energy expenditure with a root mean square error of 0·42 MET, 22·2 % lower than using a hip accelerometer alone
Liu *et al.*, 2015	Self-monitoring (physical activity and energy expenditure), self-control (eating, physical activity), optimise goal setting	Gesture recognition and real-time analytics with personalised micro-interventions (1 subject)	To recognise physical activity and provide health feedback	Android phone with built-in accelerometer and magnetometer	Nine basic daily physical activities: walking, jogging, ascending and descending stairs, bicycling, travelling up in an elevator, travelling down in an elevator, using an escalator and remaining stationary	Achieved an average recognition accuracy of 98·0 % with a minimised energy expenditure
Liu *et al.*, 2018	Self-monitoring (eating behaviour)	Image recognition	To recognise food items	Camera on smartphones	Food type	(1) outperformed existing work in terms of food recognition accuracy (top-1: 77·5 %; top-5: 95·2 %); (2) reduced response time that is equivalent to the minimum of the existing approaches and (3) lowered energy consumption which is close to the minimum of the state of the art
Lo *et al.*, 2020	Self-monitoring (eating behaviour)	Image recognition	To estimate the portion size of food items consumed	Camera	Portion size (often commonly seen food categories including burger, fried rice, pizza, etc. Each category has twenty food models with different shape geometries and portion size)	Mean accuracy of up to 84·68 %
Lopez-Meyer *et al.*, 2010	Self-monitoring (eating behaviour)	Image recognition	To describe the detection of food intake by a support vector machine classifier trained on-time history of chews and swallows	Videotaped by a camcorder to capture subject activity	Chewing and swallowing	The highest accuracy of detecting food intake (94 %) was achieved when both chews and swallows were used as predictors
Mo *et al.*, 2012	Self-monitoring (physical activity, energy expenditure)	Gesture recognition	To estimate energy expenditure	Wireless wearable multi-sensor integrated measurement system (WIMS): two triaxial accelerometers, worn at the hip and wrist	Body motion and breathing	Under free-living conditions, WIMS correctly recognised the activity intensity level 86 % of the time
Montoye *et al.*, 2016	Self-monitoring (physical activity)	Gesture recognition	To recognise physical activity type	Oxycon Mobile portable metabolic analyser and four accelerometer-based activity monitors	Physical movement	Overall classification accuracy for assessing activity type was 66–81 % for accelerometers mounted on the hip, wrists and thigh, which improved to 73–87 % when combining similar activities into categories. The wrist-mounted accelerometers achieved the highest accuracy for individual activities (80·9–81·1 %) and activity categories (86·6–86·7 %); accuracy was not different between wrists. The hip-mounted accelerometer had the lowest accuracy (66·2 % individual activities, 72·5 % activity categories)
Päßler *et al.*, 2014	Self-monitoring (eating behaviour)	Sound recognition	To recognise chewing sounds	Microphones applied to the outer ear canal	Chewing sound	Precision and recall over 80 % were achieved by most of the algorithms
Parkka *et al.*, 2010	Self-monitoring (physical activity)	Gesture recognition	To automatically recognise the physical activity	Nokia wireless motion bands 3-D accelerometer	Physical movement	Overall accuracy was 86·6 and 94·0 % after classifier personalisation
Pouladzadeh *et al.*, 2014	Self-monitoring (energy intake)	Image recognition	To estimate food energy and nutrition	The built-in camera of smartphones or tablets	Food size, shape, colour and texture. Food portion was estimated based on the area	Accuracies in detecting single, non-mixed and mixed foods were 92·21, 85 and 35–65 %, respectively
Pouladzadeh *et al.*, 2015	Self-monitoring (eating behaviour)	Sound recognition	To estimate food energy and nutrition using a cloud-based support vector machine (SVM) method	Built-in camera of smartphones or tablets	Food size, shape, colour and texture. Food portion was estimated based on the area	By using a cloud computing system in the classification phase and updating the database periodically, the accuracy of the recognition step has increased in single food portion, a non-mixed and mixed plate of food compared with LIBSVM
Rabbi *et al.*, 2015	Self-monitoring (physical activity), self-control (physical activity), optimise goal setting	Real-time analytics with personalised micro-interventions	To automatically (1) track physical activity, (2) analyse activity and food logs to identify frequent and nonfrequent behaviours and (3) generate personalised suggestions that ask users to either continue, avoid or make small changes	Accelerometer and GPS, smartphone food logging	Four most common daily physical activities – walking, running, stationary (sitting or standing) and driving	Physical activity increased by 2·8 metabolic equivalents of task (MET) – hours per week (sd 6·8; *P* = 0·02)
Rachakonda *et al.*, 2020	Self-monitoring (energy intake)	Image recognition	To automatically detect, classify and quantify the objects from the plate of the user	A camera attached to glasses	Food type, amount, time of eating	The iLog model has produced an overall accuracy of 98 % with an average precision of 85·8 %
Sazonov *et al.*, 2010	Self-monitoring (eating behaviour)	Sound recognition	To detect acoustical swallowing	Throat microphone located over laryngopharynx	Swallowing sounds	Average weighted epoch recognition accuracy for intra-visit individual models was 96·8 % which resulted in 84·7 % average weighted accuracy in detection of swallowing events
Sazonov *et al.*, 2012	Self-monitoring (eating behaviour)	Gesture recognition	To detect periods of food intake based on chewing	Piezoelectric strain gauge sensor	Jaw movement	Classification accuracy of 80·98 % and a fine time resolution of 30 s
Sazonov *et al.*, 2016	Self-monitoring (physical activity, energy expenditure)	Gesture recognition	To describe the use of a shoe-based wearable sensor system (SmartShoe) with a mobile phone for real-time recognition of various postures/physical activities and the resulting EE	Five force-sensitive resistors (integrated into a flexible insole) and an accelerometer	Physical movement	Results showed a classification accuracy virtually identical to SVM (∼95 %) while reducing the running time and the memory requirements by a factor of > 103
Spanakis *et al.*, 2017a	Self-monitoring (eating; emotions)	Predictive analytics	Analyse individual states of a person status (emotions, location, activity, etc.) and assess their impact on unhealthy eating	Classification decision trees; hierarchical agglomerative clustering	EMA 10 times a day + ad hoc entry of lapse event	Participants were clustered into six groups based on their eating behaviour and specific rules that discriminate which conditions lead to healthy *v*. unhealthy eating
Spanakis *et al.*, 2017b	Self-monitoring (eating; emotions), self-control (eating)	Real-time analytics with personalised micro-interventions	Analyse user-specific data, highlight most discriminating patterns that lead to unhealthy eating behaviour and providing feedback (personalised warning messages before a possible unhealthy eating event)	Classification decision trees and hierarchical agglomerative clustering	EMA 6 times a day + ad hoc entry of lapse event	Participants reported on average 3·6 eating events (sd = 1·1) per day
Stein *et al.*, 2017	Self-monitoring (eating; physical activity; emotions), self-control (eating; physical activity), optimise goal setting	Real-time analytics with personalised micro-interventions	Predict dietary lapses and provide adaptive semi-individualised feedback to users regarding their eating behaviour	Used a previously used algorithm which used decision tree	Chatbot	Percentage of healthy meals increased by 31 % of total meals logged at baseline to 67 % within 21 weeks; the percentage of unhealthy meals decreased by 54 %. Users averaged 2·4 kg or 2·4 % weight loss, and 75·7 % (53/70) of users lost weight in the programme
Tao *et al.*, 2018	Self-monitoring (physical activity, energy expenditure)	Gesture and image recognition	To estimate energetic expenditure	Camera, two wearable accelerometers	Physical movement, HR, exercise index, humidity and temperature	The fusion of visual and inertial data reduces the estimation error by 8 and 18 % compared with the use of visual-only and inertial sensor only, respectively, and by 33 % compared with a MET-based approach
Thomaz *et al.*, 2015	Self-monitoring (eating)	Sound recognition	Recognise eating behaviour (chewing and biting sound from ambient noises)	SVM; nearest neighbours and random forest	Wrist-worn audio recording device	Detected eating with 86·6 % accuracy
Vathsangam *et al.*, 2011	Self-monitoring (physical activity, energy expenditure)	Gesture recognition	To estimate energy expenditure during treadmill walking	Inertial measurement unit (IMU): triple-axis accelerometer, triaxial gyroscopes	Physical movement	Combining accelerometer and gyroscope information leads to improved accuracy compared with using either sensor alone
Vathsangam *et al.*, 2014	Self-monitoring (physical activity, energy expenditure)	Gesture recognition	To detect physical activity using different features	Phone-based triaxial accelerometer	Physical movement	Feature combinations corresponding to sedentary energy expenditure, sedentary heart rate and sex alone resulted in errors that were higher than speed-based models and nearest-neighbour models. Size-based features such as BMI, weight and height produced lower errors. Weight was the best individual descriptor followed by height.
Walker *et al.*, 2014	Self-monitoring (eating behaviour)	Sound recognition	To automatically detect ingestion	Throat microphone located over laryngopharynx	Eating sound	> 94 % of ingestion sounds are correctly identified with false-positive rates around 9 % based on 10-fold cross-validation
Wang *et al.*, 2019	Self-monitoring of weight loss progress	Predictive analytics	Predict weight loss based on socio temporal context	Linear regression; stochastic gradient descent (SGD)	Secondary data	Weight loss can be predicted based on temporal-social information
Yunus *et al.*, 2019	Self-monitoring (energy intake)	Image recognition	To automatically estimate food attributes such as ingredients and nutritional value	Existing image datasets	Food type and portion	Results showed the top 1 classification rate of up to 85 %
Zhang *et al.*, 2017	Self-monitoring (eating behaviour)	Gesture and image recognition	To detect eating	Wrist-worn sensor (Microsoft Band 2-accelerometer and gyroscope) and an HD webcam camera	Eating and non-eating gestures	Results showed a correlation between feeding gesture count and energetic intake in unstructured eating (*r* = 0·79, *P*-value = 0·007)
Zhang *et al.*, 2018	Self-monitoring (physical activity)	Wi-Fi signal recognition	To recognise general physical activity	The software platform, a signal transmitter and a signal receiver	Wi-Fi signal	Results showed a recognition rate of the general presence of physical activity of 99·05 %, an average recognition rate of 92 % when detecting four common classes of activities
Zhou *et al.*, 2019	Self-monitoring (physical activity)	Predictive analytics	Predict exercise lapse	SVM; logistic regression	Secondary data	Discontinuation prediction score (DiPS) makes accurate predictions on exercise goal lapse based on short-term data. The most predictive features were steps and physical activity intensity
Zhou *et al.*, 2020	Self-monitoring (physical activity), self-control (physical activity), optimise goal setting	Real-time analytics with recommendations	Adaptively compute personalised step goals that are predicted to maximise future physical activity for each participant based on all the past steps’ data and goals of each participant	Behavioural analytics algorithm (BAA)	Phone accelerometer	Participants in the intervention group had a decrease in mean (sd) daily step count of 390 (490) steps between run-in and 10 weeks, compared with a decrease of 1350 (420) steps among control participants (*n* 30; *P* = 0·03). The net difference in daily steps between the groups was 960 steps (95 % CI 90, 1830 steps)

### Functions of artificial intelligence in self-regulation of weight loss-related behaviours

We categorised the included articles into three AI applications, namely *machine perception* (*n* 50), *predictive analytics* only (*n* 6)^(47,55,82,105,107,111)^ and *real-time analytics with personalised micro-interventions* (*n* 10)^(26,49–54,107)^ (Fig. 3). Briefly, *machine perception* refers to the use of machine learning to detect, extract features, classify and interpret (recognise) information that is received through wearable/non-wearable devices – akin to our vision (camera), proprioception (gestures) and audition (sound)^(112)^. *Predictive analysis* refers to the use of historic data and statistical methods (e.g., data mining and modelling) to predict future events. Studies on predictive analytics focused on building predictive models based on behaviour data (eating and exercise), nutrition, goal achievement rates, anthropometric data, perspectives (e.g., blog posts) and ecological factors to predict weight loss and behaviour lapses. *Real-time analytics* refers to the instantaneous analysis of past and present data to train, test and optimise predictive models and provide corresponding prompts of behavioural lapse risks and recommendations as micro-interventions. Only one of the studies explored the use of all three AI applications in enhancing weight loss^(51)^. A summary of the AI features, instruments/sensors used, sensing domains and their corresponding functions relevant to weight management is shown in Table 3.

**Table 3:** Summary of AI features (that uses machine learning), instruments/sensors, sensing domains and functions about weight management

AI features	Instruments/sensors	Sensing domains
Gesture recognition	Accelerometer^(26,49,50,29,48,51,52,55,61,62,65,81–92,95,96,98,99,101,103,104)^, magnetometers^(51,81,83,90,102)^ and gyroscope^(61,65,81,82,89–91,102)^ (built-into smartphones kept in front pocket or worn on the ear, wrist, hip or leg)	Inertia: arm, hand-to-mouth, jaw movement (bite detection), physical activity
	Five force-sensitive resistors^(100)^	Pressure
	Heart rate monitor (patch-type sensor or built-in smartphone/smartwatch)^(86)^, photoplethysmography^(107)^, electrocardiogram^(95)^, ventilation sensor^(96)^	Physiological: cardiovascular parameters mainly heart rate, ventilation
	Piezoelectric sensor^(56,60,70,97)^, electromyography (EMG) electrodes (temporal muscle activity during mastication)^(58,60,64)^	Swallowing movement (jaw and skin), chewing cycle and food type
	Global positioning system (GPS)^(52,87)^	Location
Image recognition	Video recorder^(63,68)^	Images of food item/group/type
	Camera^(34,66,67,74,78,79,93,94)^	Images of food size, shape, colour, portion and texture
Sound recognition	Ear microphone (worn on one’s inner ear, outer ear, wrist, neck near the jaw/throat)^(58,59,69,71,73)^	Chewing sound (bone-conducted food breakdown sounds)
Speech recognition	Microphone (smartphone in-build microphone)^(75)^	Verbal food description or nutrition label
Wireless signal recognition	Signal receiver^(109)^	Wireless signals

#### Machine perception: self-monitoring

Studies on machine perception were focused on examining the use of machine learning techniques to recognise (1) food items/groups (e.g., fruits or meat)/types (e.g., liquid or solid), (2) eating behaviours/habits (e.g., eating behaviour lapses), (3) physical activities types (e.g., aerobic and strength-training exercises)/intensity (e.g., sedentary to vigorous exercise)/habits and (4) estimate energy balance (energetic intake and output) (Table 2). The studies reported recognition accuracies ranging from 69·2 to 99·1 %. Machine recognition techniques used in the included studies were gesture (*n* 32)^(51,56,58,60–62,64,65,70,74,81,83–93,95–104)^, image (*n* 14)^(34,63,66–68,74,76,78–80,88,93,94,101)^, sound (*n* 7)^(57–59,69,71–73)^, speech (*n* 2)^(75,77)^and wireless signal (*n* 1)^(113)^ recognition. Four studies used both gesture and image recognition^(74,88,93,101)^ while one used gesture and sound recognition^(58)^. Wearable sensors were used in all the included studies on machine perception except those that used image and wireless signal recognition (which use cameras and Wi-Fi receivers). Energy intake was mostly estimated using image and speech recognition^(34,75–80)^ while the other AI recognition techniques were used to detect eating behaviours and food types. Gesture/image recognition was mainly used to detect and estimate physical activity and energy expenditure^(83,85,92,94–97,100,101)^ while the other AI techniques were used only for physical activity recognition.

#### Predictive analytics: goal setting and action planning optimisation

Six studies showed the use of AI to predict weight loss (*n* 1)^(82)^, adherence to personalised physical activity goals (*n* 2)^(55,105)^, dietary lapses (*n* 2)^(47,107)^ and episodes of emotional eating (*n* 1) (Table 2). Only one study collected primary data using the ecological momentary assessment (EMA), which was also the only one that reported a mean dietary lapse frequency of 3·5 per week. EMA refers to the ‘repeated sampling of subjects’ current behaviours and experiences in real-time, in subjects’ natural environments’. None of the studies examined the applicability of these predictive models to stimulate weight loss. The sample sizes of the included studies on predictive analytics ranged from 12 to 210, of which only 83·3 % of the studies reported their participants’ BMI. Mean BMI of these studies ranged from 22·1 to 33·6 kg/m^2^, which were higher than those studies on machine perception and hence possibly more applicable to overweight adults. 83·3 % of the articles reported mean ages that ranged from 22·1 to 55·2 years old, one study included only female participants and the proportion of females in the remaining studies ranged from 77 to 91·7 %. Two studies explicitly reported the recruitment of only adults who were overweight, which elucidates the unique weight loss trajectory in one who is overweight although it does not indicate strategies that are effective in weight loss^(47,82)^.

#### Real-time analytics and personalised micro-interventions: self-control

Ten studies evaluated the use of AI-assisted weight management interventions that instantaneously optimise prediction models for behavioural risk profiling (e.g., low, medium and high risk) and enhance behavioural self-control through adaptive and personalised messages/feedback/prompts (Table 4). The interventions were all delivered through smartphone apps, namely OnTrack (used in three of the included studies)^(48–50)^, Sweetech app^(26)^, Calfit app^(54)^, Lark’s AI health coach app^(53)^, Think Slim app^(106)^, SmartCare app^(51)^, MyBehaviour^(52)^ and one without a name. In general, the mobile app interventions used either wrist-worn activity trackers, smartphone in-built accelerometers or EMA to track one’s physical activity. Manual food logging and EMA were commonly used to track one’s dietary habits (e.g., type, amount and triggers of food intake). Resultant data were then used to train the app’s machine learning technology to recommend optimised goals and action plans for better self-control, adherence and success in weight loss and weight loss maintenance. More details on each intervention are shown in Table 4. Intervention duration ranged from 3 to 16 weeks of which 50 % of the studies reported the inclusion of run-in periods of 1–2 weeks to collect baseline user data and assess user technological uptake and adherence^(48–50,54,106)^. Of the ten studies on real-time analytics, one used Chatbots^(53)^ and five used EMA^(47–50,106)^. EMA frequency ranged from six to ten times a day and the number of EMA questions ranged from 15 to 21 questions. Common questions were on timing (e.g., morning; afternoon; night), location (e.g., home; work), emotions (e.g., sadness; boredom; stress), activity (e.g., watching television; socialising) physical state/internal cue (e.g., hunger; cravings; fatigue) and situational triggers (e.g., visual food temptation/availability). The remaining three studies collected data on step count using accelerometers and food intake using manual logging through smartphone apps.

**Table 4:** Details of studies that used real-time analytics with personalised micro-interventions (*n* 10)

Author, year	Intervention	Intervention duration; run-in period*	Subjective data	Objective data	Feasibility and acceptability	Accuracy
Everett *et al.*, 2018	Sweetech app – uses machine learning to automatically translate raw data streams originating from the patient’s mobile phone and into insights about the individual’s life habit and provides personalised, contextual, just-in-time, just-in-place, recommendations	12 weeks, NR	Demographic information, past medical history and medications. 4-item Physical Activity States of Change Questionnaire	Weight: digital body weighing scale (Bluetooth) Waist circumference: flexible measuring tape Phone accelerometer	86 % retention; Validated System Usability Scale: median 78 %; 74 % would like to use the Sweetch app; 83 % found the app easy to use; 72 % found the functions of the app well integrated, 89 % felt that most people could learn to use the app very quickly and 77 % felt confident using the app	NR
Forman *et al.*, 2018	OnTrack + dietary weight loss programme called Weight Watcher (WW) – uses machine learning algorithm to automatically build models of lapse behaviour, predict lapses before they occur and delivers micro-interventions (messages) when lapse risk is high	8 weeks; 2 weeks	Behavioural risk factors and lapse behaviour (21: affect, boredom, hunger, cravings, tiredness, unhealthy food availability, temptations, missed meals/snacks, self-efficacy (confidence), motivation, socialising (with or without food present), TV, negative interpersonal interactions, healthy food presence, cognitive load, food cues (advertisements), hours of sleep, exercise, alcohol consumption, planning food intake, time of the day	Height and weights: calibrated scale and stadiometer	85·1 % completed; 70·15 % opened risk alerts; Technology Acceptance Model Scales (TAMS): M = 6·14, sd = 1·58 (app was easy to use); minimal technical issues (M = 2·91 out of 7, sd = 1·24); Participants rated the app as moderately useful (M = 4·64, sd = 1·58) and enjoyable (M = 4·37, sd = 1·62), with a somewhat positive behavioural intention to use (M = 4·48, sd = 1·86)	72 % accuracy, 70 % sensitivity and 72 % specificity, 80 % negative predictive value
Forman *et al.*, 2019a	OnTrack + WW	10 weeks; 2 weeks	Behavioural risk factors and lapse behaviour (seventeen potential lapse triggers, i.e., affect, boredom, hunger, cravings, tiredness, unhealthy food availability, temptations, missed meals/snacks, self-efficacy, socialising, watching TV, negative interpersonal interactions, cognitive load, food cues/advertisements, hours of sleep, alcohol consumption, and planning food intake. Time of day, automatically measured, served as an 18th trigger.)	Weight: Yumani Smart Scale (Bluetooth)	64·4 % completed; 46·9 % opened risk alerts; TAMS: M = 4·70, sd = 1·52	69·2 % sensitivity; 83·8 % specificity
Forman *et al.*, 2019b	AI-optimised interventions include individually optimised (i.e., at each of the 24 intervention points, participants receive the intervention with the highest reward score for them so far, except when the system is ‘exploring’) or group-optimised (i.e., interventions are assigned based on the highest possible total reward scores, across all interventions assigned, given a predetermined amount of total intervention time across all participants for the day)	16 weeks; NR	Energy intake: participants logged all food and beverages using the Fitbit mobile phone application	Weigh: Yumani Smart Scale (Bluetooth) Physical activity: measured in minutes of moderate-to-vigorous physical activity (MVPA) using a wrist-worn activity tracker	A short survey of coaches: the portal was easy to use (M = 3·33 out of 4) and able to effectively carry out the remote coaching (M = 3·33 out of 4); 76·5 % reported that the contact frequency was satisfactory	NR
Liu *et al.*, 2015	SmartCare – an energy-efficient long-term physical activity tracking system that follows users’ physical activity habits and gives personalised quantitative health assessment and health regime suggestion	4 weeks; NR	Users’ daily physical activities and body type: nine basic daily physical activities: walking, jogging, ascending and descending stairs, bicycling, travelling up in an elevator, travelling down in an elevator, using an escalator, and remaining stationary	Smartphone built-in accelerometer and magnetometer	NR	98 % accuracy in physical activity recognition
Rabbi *et al.*, 2015	MyBehaviour – (1) uses a combination of automatic and manual logging to track physical activity (e.g., walking, running, gym), user location, and food, (2) automatically analyse activity and food logs to identify frequent and nonfrequent behaviours and (3) generate personalised suggestions that ask users to either continue, avoid or make small changes to existing behaviours to help users reach behavioural goals	3 weeks, NR	Activity tracking and manual food logging either by selecting food items from a database or directly input energy information from nutrition labels. (Users can take photos of food as reminders to input energy intake)	Accelerometer and GPS	According to the suggestion-rating survey, participants in the experimental group had a significantly higher intention to follow personalised suggestions than those in the control group in following generic suggestions	NR
Goldstein *et al.*, 2020	Two OnTrack versions – OnTrack-short (OT-S) (8 lapse trigger questions at each EMA survey) and OnTrack-long (OT-L) (17 lapse triggers questions at each EMA survey). When an EMA survey was completed, the algorithm classified responses as no risk (when a prediction was ‘no lapse’), low risk (probability of lapse > 40 %), medium risk (probability of lapse between 40 and 70 %) or high risk (probability of lapse > 70 %)	10 weeks; 2 weeks	Seventeen lapse triggers: affect, sleep, fatigue, hunger, motivation to adhere to a diet, cravings, boredom, temptation, cognitive load, confidence, socialising, television, negative interpersonal interactions, presence of tempting foods, food advertisements, planning food, alcohol, time/	NR	84·3 % completed; 65·4 % average EMA survey adherence in OT-S and 60·5% in OT-L	79·8% accuracy of lapse prediction (79·7% in OT-S *v*. 79·9% in OT-L); 74·5% sensitivity in OT-S *v*. 77·7% in OT-L; 83·1 % specificity (84·4% in OT-S *v*. 81·7% in OT-L)
Spanakis *et al.*, 2017b	Think Slim – uses machine learning to predict unhealthy eating behaviour and allow users to report potential unhealthy eating promoting factors (emotions, activities, etc.). Emphasis is given to providing feedback before possible unhealthy eating events (i.e., warn users in the appropriate time manner using a classification algorithm) and to construct groups of eating behaviour profiles (using a clustering algorithm)	8 weeks; 1 week	Fifteen lapse triggers: date, food craving, seven emotions each measured on ten-point VAS scale (worried, angry/annoyed, stressed/tense/relaxed/at ease. Cheerful/happy, sad/depressed, bored), specific craving, location, activity, specific eating, thoughts regarding eating, food intake image/	NR	70·5 % completed	NR
Stein *et al.*, 2017	Lark’s AI health – uses machine learning to power a Chatbot that mimic health professionals’ empathetic health counselling	16 weeks; NR	Weight loss, meal quality, physical activity and sleep data were collected through user input Data points were user-entered values for age, gender, height, weight, dietary intake, with self-reported anthropometric data and Web-reported diet intake/	Sleep and physical activity, partly through automatic detection by the user’s mobile phone. User engagement was assessed by duration and amount of app use	44·0 % active users by end of the intervention; In-app user trust survey: average scores for satisfaction, disappointment if not offered and health outcome were 7·9, 8·3 and 6·73	NR
Zhou *et al.*, 2020	CalFit app – mobile phone app which delivers daily step goals using push notifications and allows real-time physical activity monitoring	10 weeks; 1 week	Socio-demographic information, self-reported medical history, Barriers to Being Active Quiz (twenty-one questions on a ten-point Likert scale on seven sub-areas: lack of time, social influence, lack of energy, lack of willpower, fear of injury, lack of skill, and lack of resources), International Physical Activity Questionnaire – Short Form/	Phone accelerometer	77·5 % retention	NR

NR, not reported; IMU, inertial measurement unit.

* Included within intervention;

Three studies^(26,53,54)^ focused on only improving physical activity, four studies focused on only improving dietary behaviours^(48–50,106)^ and three studies^(29,51,52)^ focused on both. All five studies^(29,48,49,50,53)^ on *dietary lapse* prevention reported percentage increases in dietary adherence, but only one study reported statistically significant results (*P* < 0·05), suggesting mixed findings^(50)^. Two of the three studies on preventing *exercise lapses* reported significant (*P* < 0·05) increases in step count and metabolic equivalent task^(26,53)^. This could be attributed to the personalisation of goals that were coherent with each users’ lifestyle habits based on the information retrieved from their calendar apps (indicates availability for exercise) and health app (indicates activity patterns)^(26)^. Weight loss outcomes ranged from an average of 2·4 –4·7 %^(29,48–50,106)^ of which only two were statistically significant (*P* < 0·05)^(26,50)^. Three studies reported the use of Bluetooth enabled weighing machines that synchronise weight data to the users’ phone apps, while the rest used manually-input weight.

Sixty percentage of the studies were randomised controlled trials, while the rest adopted observational and quasi-experimental designs. One study only recruited adults who were overweight, while the rest also included healthy adults^(106)^. The study sample sizes ranged from 8 to 181 participants, with mean ages ranging from 28·3 to 56·6 years old, 47–86·0% of females and a mean BMI of 27·3–37·0 kg/m^2^. Three studies reported model accuracies ranging from 69·2 to 83·8% in predicting dietary lapses, which is lower than those in the studies on machine perception^(48–50)^. This could be due to the inclusion of volatile complex human behavioural factors such as dietary lapse triggers into the prediction models that could have affected the model accuracies. Retention/completion rate ranged from 44 to 86% in eight of the nine studies, indicating varying levels of adherence^(26,48,49,50,53,54,106)^. Five studies assessed user acceptability/satisfaction using short surveys and validated instruments, namely Technology Acceptance Model Scales and Validated System Usability Scale^(26,29,49,50,53)^. However, the cut-off score to indicate acceptable acceptability/satisfaction was unclear.

### Machine learning techniques

Classifiers used included decision trees (*n* 5)^(51,64,86,99,106)^, random forests (*n* 8)^(47,49,50,56,72,74,90,94)^, rotational forests (*n* 1)^(81)^, Bayesian (*n* 8)^(47,49,50,73,82,84,86,110)^, k-nearest neighbour (*n* 5)^(86,85,88,91,103)^, clustering (*n* 1)^(106)^ and support vector machines (*n* 14)^(60,68,70,71,73,78,79,86,91,96,97,100,105,107)^. Deep learning classifying techniques used were convolutional neural network (*n* 7)^(65,66,76,77,80)^ of which two were region-based convolutional neural network^(34,65)^, artificial neural network (*n* 4)^(62,87,92,98)^, generalised regression neural network (*n* 1)^(95)^, probabilistic neural network^(51)^, hidden Markov model (*n* 4)^(59,69,101,109)^ and natural language processing (*n* 2)^(75,77)^. One study used reinforcement learning^(29)^, five used liner/logistic regression^(83,89,94,100,102)^ and other classifiers with more unique machine learning algorithms include multi-armed bandit^(52)^, radial basis function network^(95)^, behavioural analytics algorithm^(54)^ and Sojourn^(93)^.

## Discussion

Through this systematic scoping review, we found and included sixty-six studies that showed the potential uses of AI in regulating eating and dietary behaviours, exercise behaviours and weight loss. We conceptualise the AI use cases as (1) machine perception to enhance self-monitoring efficiency; (2) predictive analysis to optimise weight loss goal setting and action planning and (3) real-time analytics and personalised micro-interventions to prevent behavioural lapses. In general, the third themes seemed to be the most homogeneous where all studies described the use of a mobile phone app to monitor eating/dietary/exercise behaviours, optimise goal setting based on real-time data and delivery nudges/prompts to recommend a healthier behaviour. Predictive analytics was conducted on a wide variety of variables such as step count, energy intake, dietary lapse triggers, emotions and heart rate variability. It is noteworthy that we only found six studies that focused only on predictive modelling which could explain the heterogeneity. Machine perception was the most diverse with various recognition techniques that could be used to estimate energy intake and output. However, the accuracy of recognition technology and tracking device (e.g., in recognising food items and tracking heart rates), ease of data collection (e.g., syncing from various devices to a common data storage server for computing), degree of automaticity (i.e., risk of privacy infringement), user uptake (i.e., how adherent are the users to question prompts or machine-generated recommendations), machine learning modules (e.g., steps to prepare and analyse data and selecting the most suitable model for different datasets) and the comprehensiveness of such techniques (e.g., the number of food types that can be recognised) remains challenging. This hinders the practical implementation of AI into weight management programmes in a free-living condition, which could explain why most of the included studies are at the machine perception stage and only ten are real-life use cases for weight management. Readers should note that heterogeneity tests such as Q and I^2^ were not conducted and the aforementioned observation was derived iteratively through perusal.

Participants in the studies on real-time analytics and micro-interventions were generally older (seven of eight studies reported mean age of 40–57 years old) and had a higher BMI (27–37 kg/m^2^) than the other included studies. While variables such as gender/sex/are well-known to influence the outcomes of weight management programmes due to differences in body image^(111)^, food intake choices^(112)^, self-monitoring and self-control^(113)^, we did not find studies that examined such differences. Future studies could include a subgroup analysis based on gender to identify gender-specific target variables that could enhance weight management outcomes. While all studies ascertained the benefits of AI in facilitating behavioural self-regulation, only two out of ten interventional studies showed statistically significant weight loss post-intervention. This could be due to the difference in intervention effects on a general compared with an overweight population^(114,115)^. Another reason could be due to the short interventional programme that lasted from 3 to 16 weeks, where clinically significant weight loss (> 5 % of initial body weight) is normally observed between 6–9 months post-intervention^(116)^. On the other hand, mixed findings could also be attributed to an underpowered sample size of 43 and 55 in the studies that showed significant weight loss results as compared with the rest that ranged from 52 to 181^(29,48,49,50)^. It is also possible that micro-interventions in the form of prompting affect different behaviours differently. For example, increasing physical activity may require prompts/reminders/cues to motivate an action while such prompts could have a counter-productive effect on reducing unhealthy eating as it cues the action of unhealthy eating^(117)^. Therefore, although we recognise the potential of AI in enhancing the completeness and convenience of behaviour change self-monitoring and self-control, its additional efficiency cannot be established as yet. Moreover, the majority of the studies were on machine perception while only ten were on real-time analytics with micro-interventions. This suggests that we are still in the infancy stage of applying AI on self-regulating weight loss-related behaviours as studies are still focused on building accurate and valid behaviour self-monitoring systems before testing its effectiveness in predicting and promoting weight loss.

### Machine perception

One obvious advantage of using wearable sensors for machine perception is its potential to enhance the completeness and accuracy of data collection as it reduces respondents’ self-reporting burden, a contributing factor of underreporting shown in up to 30 and 50 % of adults of normal and overweight^(58,110)^. This is commonly achieved through the automatic collection of objective behavioural data, eliminating the common barriers of adherence such as poor motivation, time constraints and negative moods^(118)^. However, none of the studies on machine perception evaluated its effects on weight loss nor behaviour change and most of the studies did not assess the accuracy of food energy estimations. This could be due to the focus on building an accurate and reliable machine perception system before assessing its validity on specific weight-related estimations. Nevertheless, studies have shown that off-loading the need for manual logging (e.g., keeping a food diary, taking pictures and scanning barcodes) reduces user burden and increases self-monitoring adherence^(119,120)^. Of note, research has shown that the frequency rather than accuracy of self-monitoring is more significant in weight loss^(121)^. Future studies could examine the efficiency and accuracy of triangulating gesture data with image and sound in self-monitoring for weight loss and actual weight loss.

Several limitations were reported including the lower accuracy of classifiers trained at a group rather than individual level^(62,110)^ and assessing in a laboratory rather than free-living conditions^(65,84,95)^. Food recognition techniques by detecting chewing and swallowing gestures may be accurate enough to discriminate between hard and soft food items but not the exact food type especially for liquids that do not need chewing^(64,70,78)^. This would affect the accuracy of energy intake estimations and non-optimal recommendations were given. In terms of usability, the use of certain wearable devices such as placing electrodes over one’s skin surface for electromyography may not be comfortable and applicable in a free-living condition. Some of the devices also required the user to switch them on and off before and after an eating episode, placing a certain amount of burden on the users. Physical activity may also be misclassified when one performs different types of exercises within the same assessment time frame^(88,93)^. Lastly, sample sizes were small and were comprised of mostly healthy young adults and hence models may not be representative of the entire population, although the data points collected were enough to develop an accurate model^(89,93,97,98,104)^. Future studies could take note of these limitations and address them when possible.

### Predictive analytics

Positive dietary outcome expectations have been shown to significantly correlate with body fat loss^(122)^, weight loss and weight loss maintenance in obese individuals^(123)^. Studies included in this category predicted weight loss based on self-reported or accelerometer-measured exercise intensity (e.g., step count and duration), self-reported diet type (i.e., fat content and food items), the researcher measured anthropometrics, adherence to counselling interventions and socio-demographic profile (i.e., age and sex). Other predictors include weight energy consumption^(124,125)^, initial body composition (mainly fat percentage), social interaction on social media, negatively worded emotional blog posts^(126,127)^, the historical success rate in diet and exercise goal achievement and food item consumed (eating poultry was found to be associated with better goal commitment than eating porcine). These studies used clustering, decision trees, bag of visual words approach and linguistic inquiry and word count to classify the data obtained. One study included the temporal closeness of weight loss-related blog posts (i.e., timestamp) and frequency of virtual social interaction (e.g., commenting on friends’ posts) into the predictive models to improve the accuracy of weight loss prediction^(127)^. Another study developed an algorithm based on the utility-maximising framework to consider the irrationalities in human behaviour change in its weight loss predictive model^(84)^. The inclusion of such behavioural concepts could inform the future development of predictive models of public health nutrition and weight loss.

However, despite the strong influence of situational and environmental factors on behavioural self-regulation, only one study included the influence of such factors using EMA in its predictive model^(48)^. EMA has been shown to enhance the reliability and validity of data collected by reducing the risk of recall bias and reflect human responses in real-world settings^(128)^. Exercise lapses were predicted by the number of weeks one has participated in a weight loss intervention and the average daily steps in comparison to that of the previous week^(47,105)^. On the other hand, dietary lapses were predicted by food type (e.g., oil, pork, fruits) and self-reported EMA factors such as boredom, motivation, cognitive load and tempting food availability^(50,129)^. In a study on 469 overweight and obese participants who attended a behavioural weight loss programme, negative affect and social situations were identified as dietary lapse triggers at 9 months into the programme while affect, urges and situational dietary adherence were significantly associated with weight loss 12 months into the programme^(130)^. Neither affect, negative physical state, urges and temptations, time pressure, nor social situation was significantly associated with physical activity^(130)^. Suggestively, the predictors of physical activity and dietary adherence differ and future research and interventions should consider examining such differences to develop target and efficient intervention.

### Real-time analytics and micro-interventions

Three studies reported significant improvements in participants’ diet and exercise lapse prevention after undergoing a micro-intervention that involved behavioural lapse self-monitoring through smartphone app nudges/prompts^(26,50,53)^. This coincides with a study that found a 1 % decrease in the risk of exercise lapse with every additional 10 min of physical activity, suggesting that prior event/experience with self-regulation success increases the likelihood of preceding adherence^(131)^. Only two studies reported a statistically significant weight loss in participants who underwent AI-assisted weight loss intervention. The randomised controlled trial with the largest sample size (*n* 181) only found a significant interventional effect when its interaction with diet type was considered^(49)^. Concurrently, this study reported the lowest completion rate of 62·9 % as compared with the two aforementioned studies with higher completion rates of 86 %^(26)^ and 97·7 %^(50)^. Given that larger sample sizes reflect higher generalisability of results, this discrepancy suggests that interventional prompts could only be effective in inducing weight loss if the users react and adhere to the weight loss prompts and recommendations. This is especially when studies have shown that prompts and reminders could be deemed annoying and reduce app utilisation. Future studies should also note issues on legitimacy, privacy, the effort required and an ability to monitor behaviours and goals automatically in real-time^(132)^.

### Potential mechanism of how artificial intelligence can be used to improve self-regulation for weight loss and weight-related behaviour changes

**Fig. 4:**
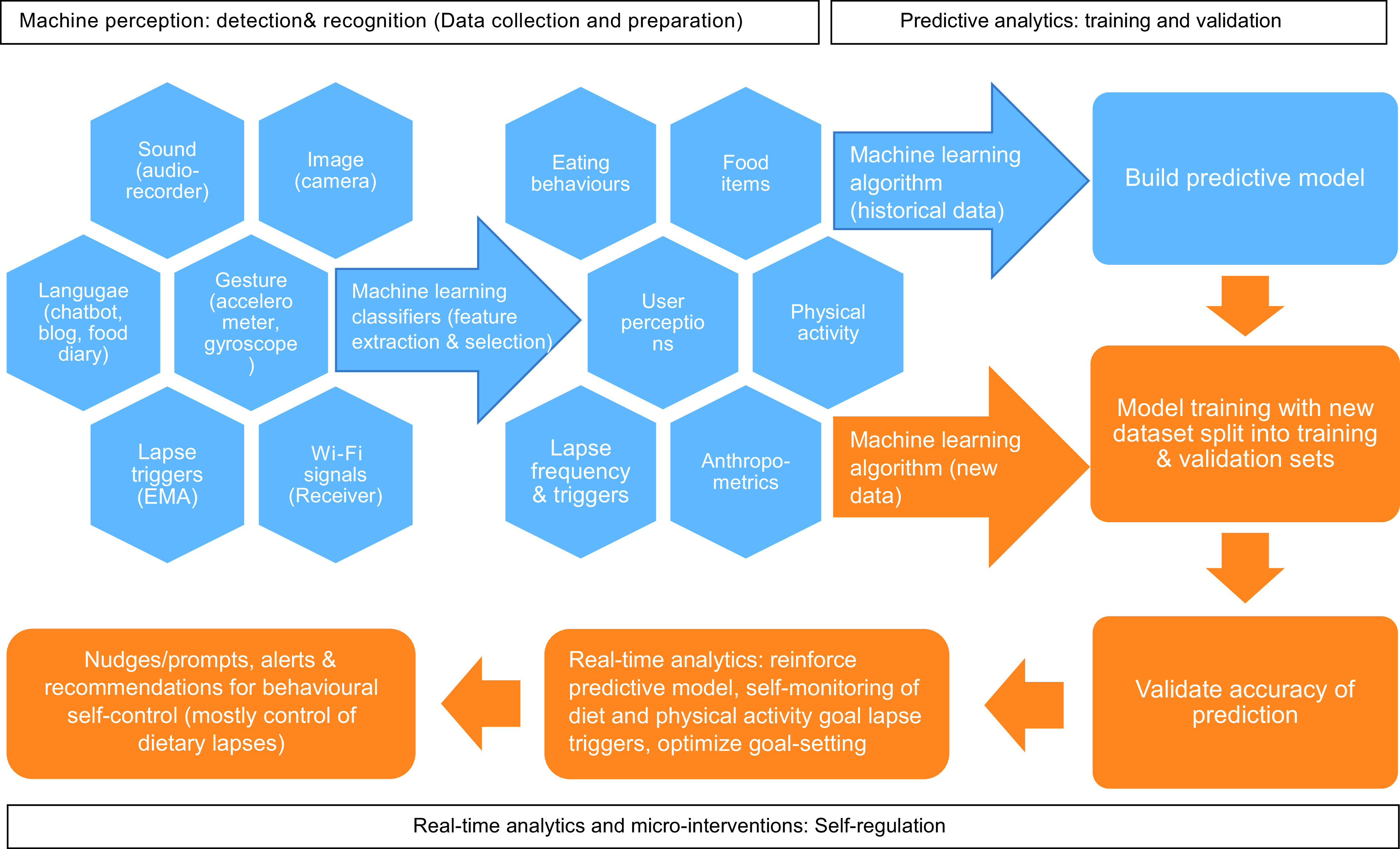
Proposed mechanism of AI-assisted self-regulation

Through this review, we highlight that a large gap in the evidence on how AI can assist in weight loss self-regulation is the lack of integration and synthesis of all three AI function categories. Therefore, we conceptualised the potential use of AI in self-regulation for weight loss based on the current findings and present it in Fig. 4. This mechanism is akin to how humans make behavioural decisions by firstly using our senses to detect and recognise certain behaviours, triggers and outcomes. Next, information is processed and learned in the brain by drawing linkages between past behaviours and current outcomes to anticipate future outcomes. Lastly, anticipations are updated based on new information while the brain decides and self-regulates behavioural outputs to achieve the desired goal^(133)^.

There are several research gaps. Firstly, intervention effectiveness should consider the influence of sex, age and comorbidities which are well-known primary predictors of body weight. Secondly, future studies on AI-assisted weight loss interventions could consider the influence of an obesogenic environment that presents one with various temptations and sets one up for self-regulation failure. Moreover, affect, habit strength and motivation have been well-established to be significant predictors of behaviour change and could be considered in future studies. It is noteworthy that data could be stored and retrieved from a cloud (on-demand data centres over the internet) or edge computing (near the source, e.g., smartphone) devices to allow machine learning algorithms to optimise and personalise existing weight loss predictive models^(134)^.

### Limitations

Firstly, the lack of Chinese database could have limited our search results on the use of AI, especially when China has been rapidly developing their technological capabilities in recent years. Future studies could examine the use of AI in studies published in other languages to facilitate further discussions on the potential of AI in self-regulation for weight loss. Next, as this scoping review aimed to present the potential of AI to enhance self-regulation for weight management, a broad and comprehensive scope of the review was needed. Therefore, although some AI applications were tested on small samples of a mixture of adults who were both healthy and overweight, such articles were included due to the consideration of feasibility that they are still at their infancy of development. Lastly, our search results could be limited to the AI applications published in academic journals and not those which have gone straight to consumer use.

## Conclusion

In summary, the current study elucidated the potential use of AI to improve weight loss through a proposed framework that includes machine perception, predictive analytics and real-time analytics with micro-interventions. However, this is contingent upon other situational, environmental and emotional factors that have to be accounted for in the AI architectures. Future studies could compare the effectiveness of AI-assisted self-regulation weight loss programmes and existing behaviour change programmes to assess the resource efficiency of AI-assisted interventions.

## Supplementary material

For supplementary material accompanying this paper visit https://doi.org/10.1017/S1368980021000598. click here to view supplementary material
