# Supplementary material for: The potential of artificial intelligence in enhancing adult weight loss: a scoping review
Source: Public Health Nutr. 2021 Feb 17;24(8):1993–2020. doi: 10.1017/S1368980021000598 (PMC8145469; doi:10.1017/S1368980021000598)
Supplement: Supplementary file 1 [file S1368980021000598sup001.docx]

Table S1: Preferred Reporting Items for Systematic reviews and Meta‐Analyses extension for Scoping Reviews (PRISMA‐ScR)

| **SECTION** | **ITEM** | **PRISMA-ScR CHECKLIST ITEM** | **REPORTED ON PAGE #** |
| --- | --- | --- | --- |
| **TITLE** | | | |
| Title | 1 | Identify the report as a scoping review. | 1 |
| **ABSTRACT** | | | |
| Structured summary | 2 | Provide a structured summary that includes (as applicable): background, objectives, eligibility criteria, sources of evidence, charting methods, results, and conclusions that relate to the review questions and objectives. | 1 |
| **INTRODUCTION** | | | |
| Rationale | 3 | Describe the rationale for the review in the context of what is already known. Explain why the review questions/objectives lend themselves to a scoping review approach. | 3-4 |
| Objectives | 4 | Provide an explicit statement of the questions and objectives being addressed with reference to their key elements (e.g., population or participants, concepts, and context) or other relevant key elements used to conceptualize the review questions and/or objectives. | 4 |
| **METHODS** | | | |
| Protocol and registration | 5 | Indicate whether a review protocol exists; state if and where it can be accessed (e.g., a Web address); and if available, provide registration information, including the registration number. | NA |
| Eligibility criteria | 6 | Specify characteristics of the sources of evidence used as eligibility criteria (e.g., years considered, language, and publication status), and provide a rationale. | 5-6 |
| Information sources* | 7 | Describe all information sources in the search (e.g., databases with dates of coverage and contact with authors to identify additional sources), as well as the date the most recent search was executed. | 5 |
| Search | 8 | Present the full electronic search strategy for at least 1 database, including any limits used, such that it could be repeated. | 5, Table S2 |
| Selection of sources of evidence | 9 | State the process for selecting sources of evidence (i.e., screening and eligibility) included in the scoping review. | 5-6 |
| Data charting process | 10 | Describe the methods of charting data from the included sources of evidence (e.g., calibrated forms or forms that have been tested by the team before their use, and whether data charting was done independently or in duplicate) and any processes for obtaining and confirming data from investigators. | 5 |
| Data items | 11 | List and define all variables for which data were sought and any assumptions and simplifications made. | 6 |
| Critical appraisal of  individual sources  of evidence | 12 | If done, provide a rationale for conducting a critical appraisal of included sources of evidence; describe the methods used and how this information was used in any data synthesis (if appropriate). | NA |
| Synthesis of results | 13 | Describe the methods of handling and summarizing the data that were charted. | 5 |
| **RESULTS** | | | |
| Selection of sources of evidence | 14 | Give numbers of sources of evidence screened, assessed for eligibility, and included in the review, with reasons for exclusions at each stage, ideally using a flow diagram. | 6 |
| Characteristics of sources of evidence | 15 | For each source of evidence, present characteristics for which data were charted and provide the citations. | 6-7 |
| Critical appraisal within sources of evidence | 16 | If done, present data on critical appraisal of included sources of evidence (see item 12). | NA |
| Results of individual sources of evidence | 17 | For each included source of evidence, present the relevant data that were charted that relate to the review questions and objectives. | Table S3 |
| Synthesis of results | 18 | Summarize and/or present the charting results as they relate to the review questions and objectives. | 6-16 |
| **DISCUSSION** | | | |
| Summary of evidence | 19 | Summarize the main results (including an overview of concepts, themes, and types of evidence available), link to the review questions and objectives, and consider the relevance to key groups. | 16-21 |
| Limitations | 20 | Discuss the limitations of the scoping review process. | 21-22 |
| Conclusions | 21 | Provide a general interpretation of the results with respect to the review questions and objectives, as well as potential implications and/or next steps. | 22 |
| **FUNDING** |  |  |  |
| Funding | 22 | Describe sources of funding for the included sources of evidence, as well as sources of funding for the scoping review. Describe the role of the funders of the scoping review. | NA |

Table S2: Search strategy for each database

| Topic |  | Search terms | Number of studies |
| --- | --- | --- | --- |
| PubMed | 1^st^ search | ("artificial intelligence"[MeSH Terms] OR "artificial intelligence" [title/abstract] OR "machine learning" [title/abstract] OR "computational intelligence" [title/abstract] OR "computer heuristics" [title/abstract] OR "expert system" [title/abstract] OR "Fuzzy Logic" [title/abstract] OR "knowledge bases" [title/abstract] OR "natural language processing" OR "neural networks" [title/abstract]) AND ("weight loss"[MeSH Terms] OR "weight loss" [title/abstract] OR "weight management" [title/abstract] OR "weight control" [title/abstract]) | 141 |
|  | 2^nd^ search | ("artificial intelligence"[All Fields] OR "machine learning"[All Fields] OR "artificial intelligence"[MeSH Terms]) AND ("weight loss"[All Fields] OR "weight management"[All Fields] OR "diet"[All Fields] OR "eating"[All Fields] OR "physical activity"[All Fields] OR "sedentary"[All Fields] OR "exercise"[All Fields] OR "weight loss"[MeSH Terms]) AND ("overweight"[MeSH Terms] OR "overweight"[All Fields] OR "overweighted"[All Fields] OR "overweightness"[All Fields] OR "overweights"[All Fields] OR "obes*"[All Fields]) | 199 |
| Embase | 1^st^ search | (((((((("artificial intelligence"[MeSH Terms] OR "artificial intelligence"[Title/Abstract]) OR "machine learning"[Title/Abstract]) OR "computational intelligence"[Title/Abstract]) OR "expert system"[Title/Abstract]) OR "Fuzzy Logic"[Title/Abstract]) OR "knowledge bases"[Title/Abstract]) OR "natural language processing"[All Fields]) OR "neural networks"[Title/Abstract]) AND ((("weight loss"[MeSH Terms] OR "weight loss"[Title/Abstract]) OR "weight management"[Title/Abstract]) OR "weight control"[Title/Abstract]) | 139 |
|  | 2^nd^ search | ('artificial intelligence' OR 'machine learning') AND ('weight loss' OR 'weight management' OR 'diet' OR 'eating' OR 'physical activity' OR 'sedentary' OR 'exercise') AND (obes* OR overweight) | 187 |
| Cochrane-Central | 1 (1^st^ search) | MeSH descriptor: [Artificial Intelligence] explode all trees | 976 |
|  | 2 | (("artificial intelligence" OR "machine learning" OR "computational intelligence" OR "computer heuristics" OR "expert system" OR "Fuzzy Logic" OR "knowledge bases" OR "natural language processing" OR "neural networks")):ti,ab,kw | 1969 |
|  | 3 | (("weight loss" OR "weight management" OR "weight control")):ti,ab,kw | 21785 |
|  | 4 | MeSH descriptor: [Weight Loss] explode all trees | 6225 |
|  | 5 | (#1 OR #2) AND (#3 OR #4) | 45 |
|  | 2^nd^ search | ('artificial intelligence' or 'machine learning') AND ('weight loss' or 'weight management' or 'diet' or 'eating' or 'physical activity' or 'sedentary' or 'exercise') AND (obes* or overweight)" (Word variations have been searched) | 20 |
| CINAHL | 1^st^ search | ("artificial intelligence" OR "artificial intelligence" OR "machine learning" OR "computational intelligence" OR "computer heuristics" OR "expert system" OR "Fuzzy Logic" OR "knowledge bases" OR "natural language processing" OR "neural networks") AND ("weight loss" OR "weight loss" OR "weight management" OR "weight control") | 33 |
|  | 2^nd^ search | TX ( 'artificial intelligence' OR 'machine learning' ) AND TX ( 'weight loss' OR 'weight management' OR 'diet' OR 'eating' OR 'physical activity' OR 'sedentary' OR 'exercise' ) AND TX ( obes* OR overweight ) | 220 |
| PsycINFO | 1^st^ search | (("artificial intelligence" or "artificial intelligence" or "machine learning" or "computational intelligence" or "computer heuristics" or "expert system" or "Fuzzy Logic" or "knowledge bases" or "natural language processing" or "neural networks") and ("weight loss" or "weight loss" or "weight management" or "weight control")).mp. [mp=title, abstract, heading word, table of contents, key concepts, original title, tests & measures, mesh] | 39 |
|  | 2^nd^ search | (('artificial intelligence' or 'machine learning') and ('weight loss' or 'weight management' or 'diet' or 'eating' or 'physical activity' or 'sedentary' or 'exercise') and (obes* or overweight)).af. | 479 |
| IEEE xplore | 1 (1^st^ search) | (("Document Title":"artificial intelligence" OR "machine learning" OR "computational intelligence" OR "computer heuristics" OR "expert system" OR "Fuzzy Logic" OR "knowledge bases" OR "natural language processing" OR "neural networks") AND "Document Title":"weight loss" OR "weight management" OR "body weight control") | 63 |
|  | 2 | (("Abstract":"artificial intelligence" OR "machine learning" OR "computational intelligence" OR "computer heuristics" OR "expert system" OR "Fuzzy Logic" OR "knowledge bases" OR "natural language processing" OR "neural networks") AND "Abstract":"weight loss" OR "weight management" OR "body weight control") | 75 |
|  | 3 | (("Document Title":"artificial intelligence" OR "machine learning" OR "computational intelligence" OR "computer heuristics" OR "expert system" OR "Fuzzy Logic" OR "knowledge bases" OR "natural language processing" OR "neural networks") AND "Abstract":"weight loss" OR "weight management" OR "body weight control") | 75 |
|  | 4 | (("Abstract":"artificial intelligence" OR "machine learning" OR "computational intelligence" OR "computer heuristics" OR "expert system" OR "Fuzzy Logic" OR "knowledge bases" OR "natural language processing" OR "neural networks") AND "Document Title":"weight loss" OR "weight management" OR "body weight control") | 63 |
|  | 5 | #1 AND #2 AND #3 AND #4 | 75 |
|  | 2^nd^ search | ((("Full Text & Metadata":'artificial intelligence' OR 'machine learning') AND "Full Text & Metadata":'weight loss' OR 'weight management' OR 'diet' OR 'eating' OR 'physical activity' OR 'sedentary' OR 'exercise') AND "Full Text & Metadata":obes* OR overweight) | 1713 |
| Scopus | 1^st^ search | ( TITLE-ABS-KEY ( "artificial intelligence" OR "machine learning" OR "computational intelligence" OR "computer heuristics" OR "expert system" OR "Fuzzy Logic" OR "knowledge bases" OR "natural language processing" OR "neural networks" ) AND TITLE-ABS-KEY ( "weight loss" OR "weight management" OR "weight control" ) ) | 465 |
|  | 2^nd^ search | ( TITLE-ABS-KEY ( "artificial intelligence" OR "machine learning" OR "computational intelligence" OR "computer heuristics" OR "expert system" OR "Fuzzy Logic" OR "knowledge bases" OR "natural language processing" OR "neural networks" ) AND TITLE-ABS-KEY ( "weight loss" OR "weight management" OR "weight control" ) ) | 1345 |
| Web of Science | 1^st^ search | (("artificial intelligence" OR "artificial intelligence" OR "machine learning" OR "computational intelligence" OR "computer heuristics" OR "expert system" OR "Fuzzy Logic" OR "knowledge bases" OR "natural language processing" OR "neural networks")) AND TOPIC: (("weight loss" OR "weight loss" OR "weight management" OR "weight control")) | 193 |
|  | 2^nd^ search | ALL FIELDS: ('artificial intelligence' or 'machine learning') AND ALL FIELDS: ('weight loss' or 'weight management' or 'diet' or 'eating' or 'physical activity' or 'sedentary' or 'exercise') AND ALL FIELDS: (obes* or overweight) | 249 |

Table S3: Study characteristics (N= 66)

|  | **Author,**  **Year** | **Country** | **Study design** | **Aim** | **Population /**  **Data source** | **Age Mean ± SD / range** | **Gender (Female)** | **Baseline BMI mean ± SD / range** |
| --- | --- | --- | --- | --- | --- | --- | --- | --- |
|  | Alshurafa 2015 | US | Experimental | To propose an algorithm based on time-frequency decomposition, spectrogram analysis of piezoelectric sensor signals, to accurately distinguish between food types, such as liquid and solid (texture), hot and cold drinks (temperatures), and hard and soft (consistencies) foods. | 20 subjects | 20-40 | 8 (40%) | NR |
|  | Amft 2008 | Switzerland | Experimental | To examine the continuous recognition of dietary activities using on-body sensors. | 4 subjects | 25-35 | 1 (25%) | NR |
|  | Amft 2009 | Switzerland | Experimental | To evaluate the prediction of food weight in individual bites using an ear-pad chewing sound sensor. | 8 subjects | 20-30 | 2 (25%) | NR |
|  | Arif 2015 | Saudi Arabia | Secondary data analysis | Classify physical activities using wearable sensors | 9 subjects | 27.2 ± 3.3 | 1 (11.1%) | 25.11 ± 2.6 |
|  | Aswani 2019 | US | Secondary data analysis of feasibility RCT | Develop a model to predict weight loss after a long period of time (e.g., 5 months) in individuals participating in a weight loss program as a function of the physical activity goals and amount of counseling using a short time-span (e.g., 15–30 days) of physical activity and weight data | 61 subjects | 55.2 ± 9.0 | 47 (77%) | NR |
|  | Aziz 2020 | Canada | Experimental | To present an experimental study to estimate energy expenditure during sitting, standing and treadmill walking using a smartwatch. | 10 subjects | 27.09 ± 5.0 | 4 (40%) | NR |
|  | Bastian 2015 | France | Experimental | To automatically discriminate between 8 activity classes (lying, slouching, sitting, standing, walking, running, and cycling) in a laboratory condition and walking the streets, running, cycling, and taking the bus in free-living conditions. | 59 subjects (lab); 20 subjects (free-living condition) | 37.3 ± 10.6; 18–39 | 59 (49.2%) | 26 subjects < 25kg/m^2^, 16 subjects 25-30 kg/m^2^, 17 subjects > 30 kg/m^2^ |
|  | Bi 2016 | China | Experimental | To present AutoDietary, a wearable system to monitor and recognize food intakes in daily life. | 12 subjects | NR | NR | NR |
|  | Bouarfa 2013 | UK | Experimental | To examine whether a single ear-worn accelerometer can be used for energy expenditure (EE) estimation under free-living conditions. | 25 subjects | 29.96 ± 4.53 | NR | 23:87 ± 4:93 |
|  | Chung 2018 | South Korea | Experimental | To present a series of protocols of designing and manufacturing a glasses-type wearable device that detects the patterns of temporalis muscle activities during food intake and other physical activities. | 10 subjects | 27.9 ± 4.3 | 5 (50%) | 21.6 ± 3.2 |
|  | Dijkhuis 2018 | The Netherlands | RCT | To investigate the possibility of automating part of the coaching procedure on physical activity by providing personalized feedback throughout the day on a participant’s progress in achieving a personal step goal. | NR | NR | NR | NR |
|  | Dobbins 2016 | UK | Experimental | To evaluate the performance of supervised machine learning in distinguishing physical activity. | 22 subjects | NR | NR | NR |
|  | Dong 2014 | US | Experimental | To describe a new method that uses a watch-like configuration of sensors to continuously track wrist motion throughout the day and automatically detect periods of eating (free-living condion). | 43 subjects | 18-50 | 35 (81.4%) | NR |
|  | Ermes 2008 | Finland | Experimental | To examine how well the daily activities and sports performed by the subjects in unsupervised settings can be recognized compared to supervised settings. | 12 subjects | NR | NR | NR |
|  | Everett 2018 | US | Observational | To calibrate the Sweetch app and determine the feasibility, acceptability, safety, and effectiveness of the Sweetch app in combination with a digital body weight scale (DBWS) in inccreasing leisure time physical activity and weight loss | 55 subjects | 55 ±10.06 | 33 (60%) | 32.6 (4.5) |
|  | Fontana 2014 | US | Experimental | To describe a novel wearable sensor system (automatic ingestion monitor, AIM) is presented for objective monitoring of ingestive behavior in free living. | 12 subjects | 26.7 ± 3.7 | 6 (50%) | 24.39 ± 3.81 |
|  | Forman 2018 | US | Observational study | To evaluate the feasibility, acceptability, and preliminary effectiveness of **OnTrack** among weight loss program (8 weeks) participants. | 43 subjects | 50.98 ± 12.72 | 37 (86%) | 35.6±5.88 |
|  | Forman 2019a | US | RCT | eEamine the additive efficacy of intervention in promoting weight loss over and above a comprehensive self-help weight loss mHealth program | 181 subjects | 46.29 ± 13.58 | 154 (85.1%) | 34.32, SD NR |
|  | Forman 2019b | US | RCT | To evaluate the feasibility and acceptability of a reinforcement learning-based weight loss intervention, and whether optimization would achieve equivalent benefit at a reduced cost compared to a non-optimized intensive intervention. | 52 subjects | 56.60 ± 13.35 | 35 (67.3%) | 34.30±5.76 |
|  | Fullerton 2017 | UK | Experimental | To successfully recognize activity and sub-category activity types through the use of multiple body worn accelerometers in a free-living environment. | 10 subjects | 23.1 ± 1.7 | 2 (20%) | 26.7 |
|  | Goldstein 2018 | US | Observational | To predict lapses and evaluate the utility of combining both group- and individual-level data to enhance lapse prediction. | 12 subjects | 38.25 ± 13.54 | 11 (91.7%) | 33.6 ±5.66 |
|  | Goldstein 2020 | US | RCT | Examined the effect of more questions per ecological momentary assessment survey on algorithm performance, app utilization, and behavioral outcomes | 121 subjects | 47.23 ± 13.39 | 102 (84.3%) | 34.52 ± 5.73 |
|  | Hedge 2018 | US | Experimental | To proposed an insole-based activity monitor—SmartStep, designed to be socially acceptable and comfortable. | 15 subjects | 25.06 | 7 (45.7%) | 25 |
|  | Hezarjaribi 2018 | US | Experimental | To develop and validate the Speech2Health, a voice-based mobile nutrition monitoring system that devises speech processing, natural language processing (NLP), and text mining techniques in a unified platform to facilitate nutrition monitoring | 30 subjects | 18-30 | NR | NR |
|  | Hossain 2020 | US | Experimental | To develop a method to detect and count bites and chews automatically from meal videos. | 28 subjects | 29.03 ± 12.20 | NR | 27.87 ± 5.51 |
|  | Hua 2020 | US | Experimental | To evaluate machine learning models for classifying nine different upper extremity exercises, based upon kinematic data captured from an IMU-based device. | 50 subjects | 21.9 ± 4.0 | 20 (40%) | 22.6 ± 2.7 |
|  | Huang 2017 | Hong Kong | Observational | Designed a pair of diet-aware glasses for diet monitoring of chewing | 7  subjects | NR | NR | 19 - 32 kg/m^2^ |
|  | Jain 2018 | India | Experimental | To proposes a descriptor-based approach for activity classification using built-in sensors of smartphones. | 16 subjects | 21-60 | NR | NR |
|  | Jiang 2020 | China | Experimental | To develop a deep model-based food recognition and dietary assessment system to study and analyze food items from daily meal images (e.g., captured by smartphone). | NR | NR | NR | NR |
|  | Juarascio 2020 | US | Observational | To test the hypothesis that momentary changes in HRV can be used to detect risk of experiencing an emotional eating episode in an ecologically valid setting using a wrist worn sensor with acceptable classification accuracy. | 21 subjects | 34.05 ± 14.41 | 18 (85.7%) | 27.79 ± 6.92 |
|  | Kang 2019 | South Korea | Experimental | To propose the most accurate method using a wireless patch-type sensor to predict the energy expenditure of physical activities. | 53 subjects | 32.1 | 10 (18.9%) | 30.2 |
|  | Kim 2015 | US | Experimental | To examine the validity of ActiGraph GT3X [ActiGraph LLC, Pensacola, FL, USA] and activPAL [PAL Technologies Ltd., Glasgow, Scotland]) for the assessment of sedentary behavior. | 11 subjects | 30.67 ± 7.24 | 3 (27.3%) | 25.36 ± 4.57 |
|  | Korpusik 2020 | US | Experimental | To present a nutrition dialogue system that automatically extracts food concepts (nutrients and caloric intake) from a user’s spoken meal description. | 37 subjects | NR | NR | NR |
|  | Kyritsis 2019 | Greece | Experimental | To present an algorithm for automatically detecting the in-meal food intake cycles using the inertial signals (acceleration and orientation velocity) from an off-the-shelf martwatch. | 12 subjects | NR | NR | NR |
|  | Lin 2012 | Taiwan | Experimental | To first to categorize physical activities with similar intensity levels, and then to construct energy expenditure regression (EER) models using neural networks in order to optimize the estimation performance. | 26 subjects | 22.18 ± 2.94 | 6 (23.1%) | 22.18 ± 2.94 |
|  | Lin 2015 | Taiwan | Experimental | To propose a depth-camera-based system for EE estimation of PA in gyms. | 21 subjects | 21 ± 1.5 | 11 (52.4%) | 21.42 ± 2.17 |
|  | Liu 2012 | US | Experimental | To present a sensor fusion method for assessing physical activity (PA) of human subjects, based on support vector machines (SVMs). | 50 subjects | 32.6 ± 9.9 | 31 (62%) | 23.2 ±4.6 |
|  | Liu 2015 | China | Observational | To introduce SmartCare, an energy-efficient long-term physical activity tracking system using smartphones. A practical energy saver and a hybrid classifier are proposed, which enable SmartCare to run over an extended period while achieving satisfactory classification accuracy. | 8 subjects | NR | NR | NR |
|  | Liu 2018 | US | Observational | 1. Develop novel deep learning-based visual food recognition algorithms to achieve the best-in-class recognition accuracy 2. Design a food recognition system | Existing image datasets | NR | NR | NR |
|  | Lo 2020 | UK | Experimental | To propose a novel vision-based method based on real-time three-dimensional (3-D) reconstruction and deep learning view synthesis to enable accurate portion size estimation of food items consumed. | NR | NR | NR | NR |
|  | Lopez-Meyer, 2010 | US | Experimental | To describe detection of food intake by a Support Vector Machine classifier trained on time history of chews and swallows. | 18 subjects | 18-57 | 7 (39%) | 28.01 ± 6.35 |
|  | Mo 2012 | China | Experimental | To present a wireless wearable multisenor integrated measurement system (WIMS) for realtime measurement of the energy expenditure and breathing volume of human subjects under free-living conditions. | 8 subjects | 25.9 ± 7.0 | 5 (62.5%) | 25.2 ± 3.7 |
|  | Montoye 2016 | US | Experimental | To compare accuracy of activity type prediction models for accelerometers worn on the hip, wrists, and thigh. | 44 subjects | 18-44 | 22 (50%) | NR |
|  | Pa¨ßler 2014 | Germany | Experimental | To evaluation of chewing sounds generated during the process of eating for food intake analysis. | 51 subjects | 34.8 | NR | NR |
|  | Parkka 2010 | Finland | Experimental | To evaluate an automatic activity-recognition system consisting of wireless motion bands and a PDA. | 7 subjects | 27 | 1 (12.5%) | NR |
|  | Pouladzadeh 2014a | Canada | Experimental | To propose a food calorie and nutrition measurement system that can help patients and dietitians to measure and manage daily food intake. | NR | NR | NR | NR |
|  | Pouladzadeh 2014b | Canada | Experimental | To present a cloud-based Support Vector Machine (SVM) method for classifying objects in cluster. | NR | NR | NR | NR |
|  | Rabbi 2015 | US | Pilot RCT | To investigate the technical feasibility of implementing an automated feedback system, the impact of the suggestions on user physical activity and eating behavior, and user perceptions of the automatically generated suggestions. | 17 subjects | 28.3 ± 6.96 | 8 (47%) | NR |
|  | Rachakonda 2020 | US | Experimental | To propose a food monitoring system and deep learning model for edge computing platforms which can automatically detect, classify and quantify the objects from the plate of the user. | NR | NR | NR | NR |
|  | Sazonov 2010 | US | Experimental | To propose and compare two methods of acoustical swallowing detection from sounds contaminated by motion artifacts, speech and external noise. | 20 subjects | NR | NR | NR |
|  | Sazonov 2012 | US | Experimental | To present a simple sensor system and related signal processing and pattern recognition methodologies to detect periods of food intake based on non-invasive monitoring of chewing. | 20 subjects | 18-57 | 9 (45%) | 29.0 ± 6.4 |
|  | Sazonov 2016 | US | Experimental | To describe the use of a shoe-based wearable sensor system (SmartShoe) with a mobile phone for real-time recognition of various postures/physical activities and the resulting EE. | 15 subjects | 26.34 | 9 (47.4%) | 25.12 |
|  | Spanakis 2017a | US | Observational | To analyze individual states of a person status (emotions, location, activity, etc.) using EMA and assess their impact on unhealthy eating. | 143 subjects 43 healthy | overweight: 31.2(10.0) healthy:32.1(10.6) | overweight: 87.7% healthy:88.4% | overweight: 30.3(4.3) healthy: 22.1(1.5) |
|  | Spanakis 2017b | US | RCT | To provide and test the effectiveness of a CBT-based EMI. | 100 subjects | Range: 18 to 60 years | NR | NR |
|  | Stein 2017 | US | Observational | To promote weight loss and other health behaviors related to diabetes prevention through automatic health coaching | 70 subjects | 47 ±1.89 | 52 (74.5%) | 37 ±1.40 |
|  | Tao 2017 | UK | Experimental | To present a method for estimating calorific expenditure from combined visual and accelerometer sensors by way of an RGB-Depth camera and a wearable inertial sensor | 10 subjects | 27.2 ± 3.8 | 3 (30%) | 23.7 ± 2.8 |
|  | Thomaz 2015 | US | Experimental | To describe results from a feasibility study conducted in-the-wild where eating activities were inferred from ambient sounds captured with a wrist-mounted device | 20 subjects | 21-55 | 6 (30%) | NR |
|  | Vathsangam 2011 | US | Experimental | To describe an experimental study to estimate energy expenditure during treadmill walking using a single hip-mounted inertial sensor (triaxial accelerometer and triaxial gyroscope). | 7 subjects | 29 ± 6 | 4 (57.1%) | 24 ± 8 |
|  | Vathsangam 2014 | US | Experimental | To present a set of algorithms that extend previous work to include an arbitrary number of anthropometric descriptors. | 34 subjects | 26 ± 4 | 9 (26.5%) | 23.0 ± 1.6 |
|  | Walker 2014 | US | Experimental | To present an overview of a prototype automated ingestion detection (AID) process implemented in a health monitoring system (HMS). | 7 subjects | NR | NR | NR |
|  | Wang 2010 | Australia | Experimental | To investigate the benefits of automatic gait analysis approaches including step-by-step gait segmentation and heel-strike recognition of the accelerometry signal in classifying various gradients. | 12 subjects | 27 ± 3.8 | 4 (33.3%) | 21.6 |
|  | Yunus 2019 | US | Experimental | To propose a novel system to automatically estimate food attributes such as ingredients and nutritional value by classifying the input image of food. | NR | NR | NR | NR |
|  | Zhang 2017 | US | Experimental | To present results on three experiments: highly structured (participants pretending to eat), in-lab structured with confounding activities (participants eating while performing other scripted activities), and unstructured overeating (participants induced to overeat while watching television and eating their favorite foods). | 10 subjects | 24.2 | 1 (10%) | NR |
|  | Zhou 2018 | US | RCT | To evaluate efficacy of an automated mobile phone–based personalized and adaptive goal-setting intervention as compared with an active control with steady daily step goals of 10,000. | 64 subjects | 41.1 ± 11.3 | 53 (82.8%) | 27.3 ± 6.1 |
|  | Zhou 2019 | US | Secondary data analysis of a 3-arm RCT | To develop and test adherence prediction models using objectively measured physical activity data | 210 subjects | 52.4 ± 11.0 | 100% | 18.5 – 43.0 |

*Note:* SD=standard deviation; BMI=body mass index; NR=not reported; RCT=randomized controlled trial
